# Supplementary material for: New Hydroxydecanoic Acid Derivatives Produced by an Endophytic Yeast Aureobasidium pullulans AJF1 from Flowers of Aconitum carmichaeli
Source: Molecules. 2019 Nov 8;24(22):4051. doi: 10.3390/molecules24224051 (PMC6891652; doi:10.3390/molecules24224051)
Supplement: Supplementary file 1 [file molecules-24-04051-s001.pdf]

Supporting information

New hydroxydecanoic acid derivatives produced by an endophytic yeast *Aureobasidium pullulans* AJF1 from flowers of *Aconitum carmichaeli*

Hyun Gyu Choi [1], Jung Wha Kim [1], Hyukjae Choi [2], Ki Sung Kang,[3] and Sang Hee Shim [1,*]

[1] College of Pharmacy, Duksung Women's University; chg---@hanmail.net (H. G. C.); jwkim7317@gmail.com (J.W.K.)
[2] College of Pharmacy, Yeungnam University; h5choi@yu.ac.kr
[3] College of Korean Medicine, Gachon University; kkang@gachon.ac.kr

List of Figures

Compounds 1-3

Figure 1. ^{1}H-NMR spectrum of compound **1** (500 MHz, $CDCl_3$)

Figure 2. ^{13}C-NMR spectrum of compound **1** (125 MHz, $CDCl_3$)

Figure 3. ^{1}H-^{1}H COSY spectrum of compound **1** (500 MHz, $CDCl_3$)

Figure 4. HMQC spectrum of compound **1** (500/125 MHz, $CDCl_3$)

Figure 5. HMBC spectrum of compound **1** (500/125 MHz, $CDCl_3$)

Figure 6. (+)HRFABMS spectrum and value of compound **1**

Figure 7. ^{1}H-NMR spectrum of compound **2** (500 MHz, $CDCl_3$)

Figure 8. ^{13}C-NMR spectrum of compound **2** (125 MHz, $CDCl_3$)

Figure 9. ^{1}H-^{1}H COSY spectrum of compound **2** (500 MHz, $CDCl_3$)

Figure 10. HMQC spectrum of compound **2** (500/125 MHz, $CDCl_3$)

Figure 11a. HMBC spectrum of compound **2** (500/125 MHz, $CDCl_3$)

Figure 11b. HMBC spectrum of compound **2** (300/75 MHz, $CDCl_3$)

Figure 12. (+)HRFABMS spectrum and value of compound **2**

Figure 13. ^{1}H-NMR spectrum of compound **3** (500 MHz, $CDCl_3$)

Figure 14. ^{13}C-NMR spectrum of compound **3** (125 MHz, $CDCl_3$)

Figure 15. ^{1}H-^{1}H COSY spectrum of compound **3** (500 MHz, $CDCl_3$)

Figure 16. HMQC spectrum of compound **3** (500/125 MHz, $CDCl_3$)

Figure 17. HMBC spectrum of compound **3** (500/125 MHz, $CDCl_3$)

Figure 18. (+)HRFABMS spectrum and value of compound **3**

Acid hydrolysis products

Figure 19. ^{1}H-NMR spectrum of compound **2a** (300 MHz, $CDCl_3$)

Figure 20. ^{1}H-NMR spectrum of compound **2b** (300 MHz, $CDCl_3$)

Figure 21. ^{13}C-NMR spectrum of compound **2b** (75 MHz, $CDCl_3$)

Figure 22. ^{1}H-NMR spectrum of compound **3d** (300 MHz, $CDCl_3$)

Figure 23. ^{13}C-NMR spectrum of compound **3d** (75 MHz, $CDCl_3$)

Acetonide products (1a, 2c-d, 3c)

Figure 24. ^{1}H-NMR spectrum of compound **1a** (300 MHz, $CDCl_3$)

Figure 25. ^{13}C-NMR spectrum of compound **1a** (75 MHz, $CDCl_3$)

Figure 26. ^{1}H-NMR spectrum of compound **2c** (300 MHz, $CDCl_3$)

Figure 27. ^{13}C-NMR spectrum of compound **2c** (75 MHz, $CDCl_3$)

Figure 28. ^{1}H-NMR spectrum of compound **2d** (300 MHz, $CDCl_3$)

Figure 29. ^{13}C-NMR spectrum of compound **2d** (75 MHz, $CDCl_3$)

Figure 30. ^{1}H-NMR spectrum of compound **3c** (300 MHz, $CDCl_3$)

Figure 31. ^{13}C-NMR spectrum of compound **3c** (75 MHz, $CDCl_3$)

Mosher's ester

Figure 32. ^{1}H-NMR spectrum of (*S*)-MTPA ester of **1** (500 MHz, Pyridine-d_5)

Figure 33. ^{1}H-NMR spectrum of (*R*)-MTPA ester of **1** (300 MHz, Pyridine-d_5)

Figure 34. ^{1}H-NMR spectrum of (*S*)-MTPA ester of **2** (300 MHz, Pyridine-d_5)

Figure 35. ^{1}H-NMR spectrum of (*R*)-MTPA ester of **2** (300 MHz, Pyridine-d_5)

Figure 36. ^{1}H-NMR spectrum of (*S*)-MTPA ester of **3d** (300 MHz, Pyridine-d_5)

Figure 37. ^{1}H-NMR spectrum of (*R*)-MTPA ester of **3d** (300 MHz, Pyridine-d_5)

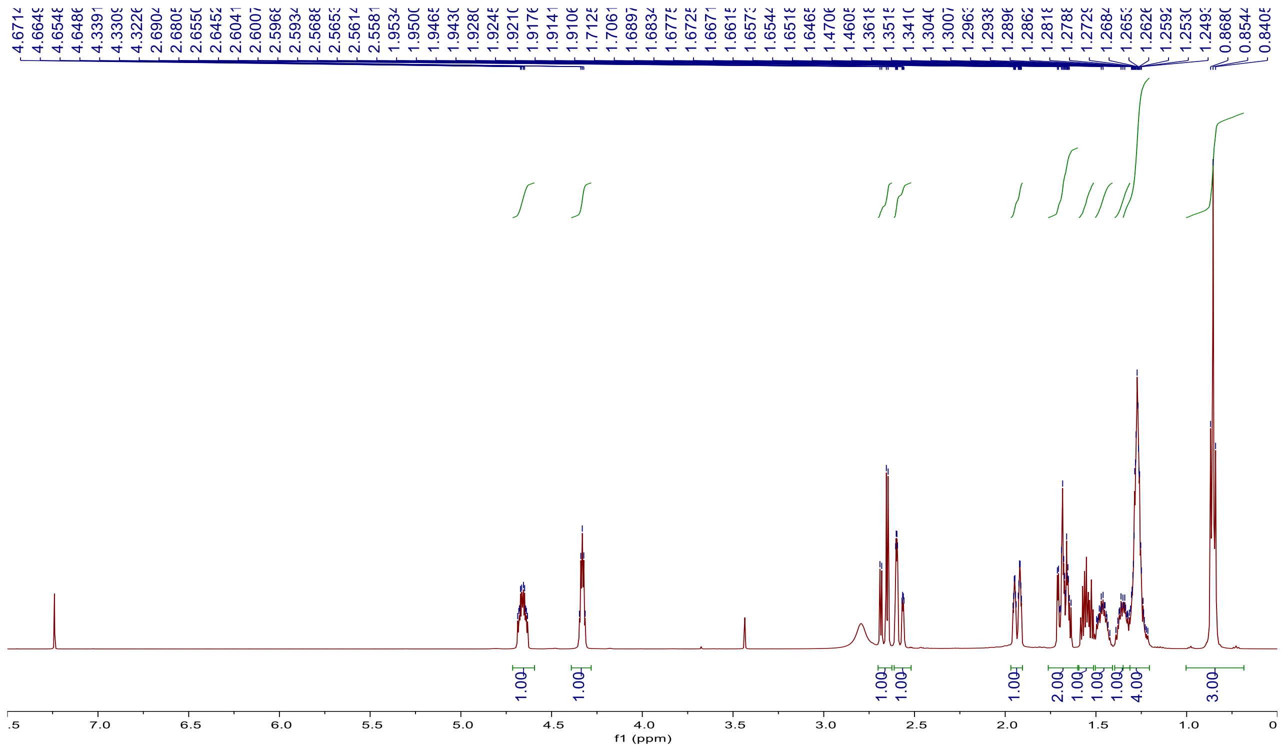

Figure 1. ^{1}H-NMR spectrum of compound **1** (500 MHz, $CDCl_3$)

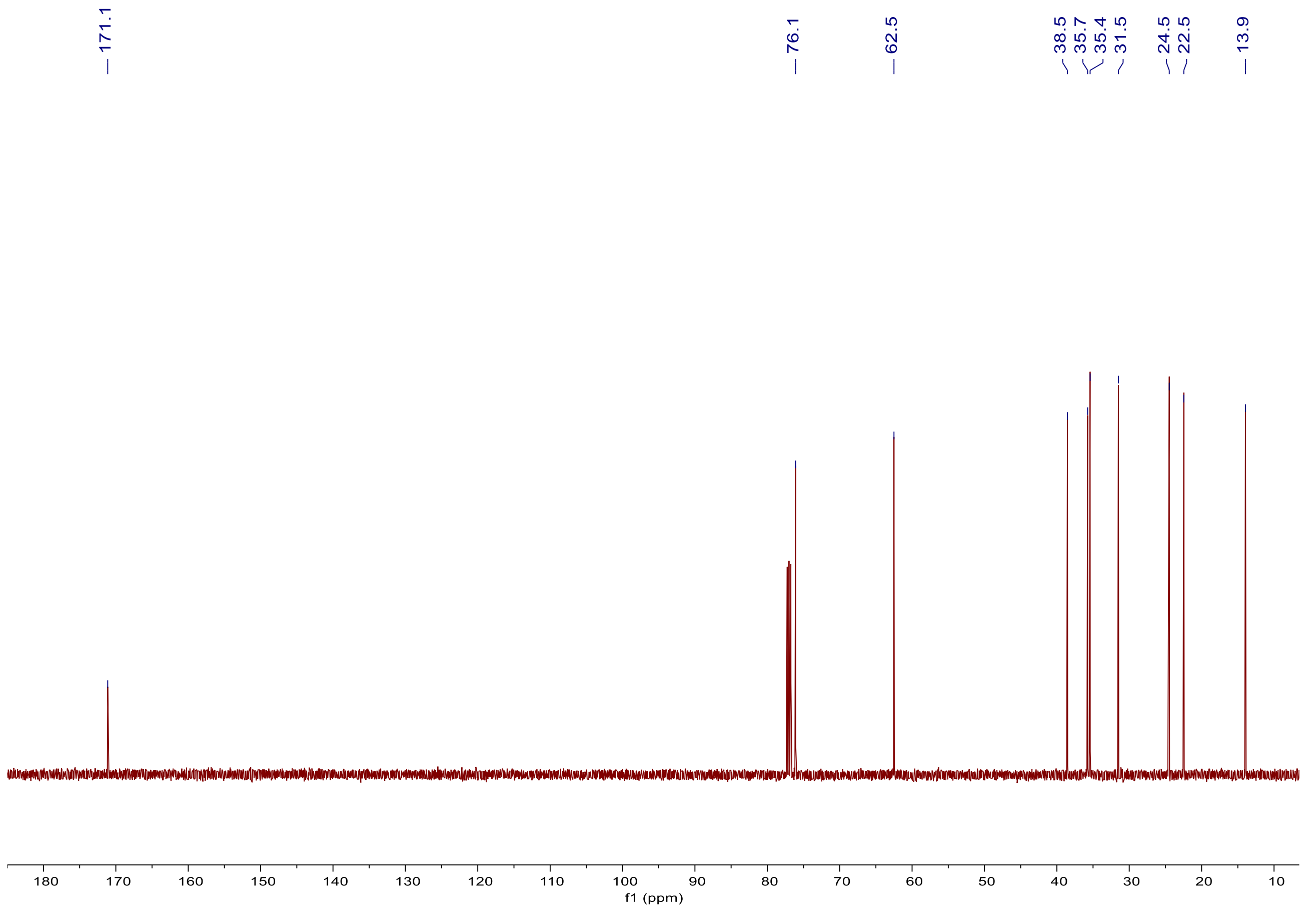

Figure 2. ^{13}C-NMR spectrum of compound **1** (125 MHz, $CDCl_3$)

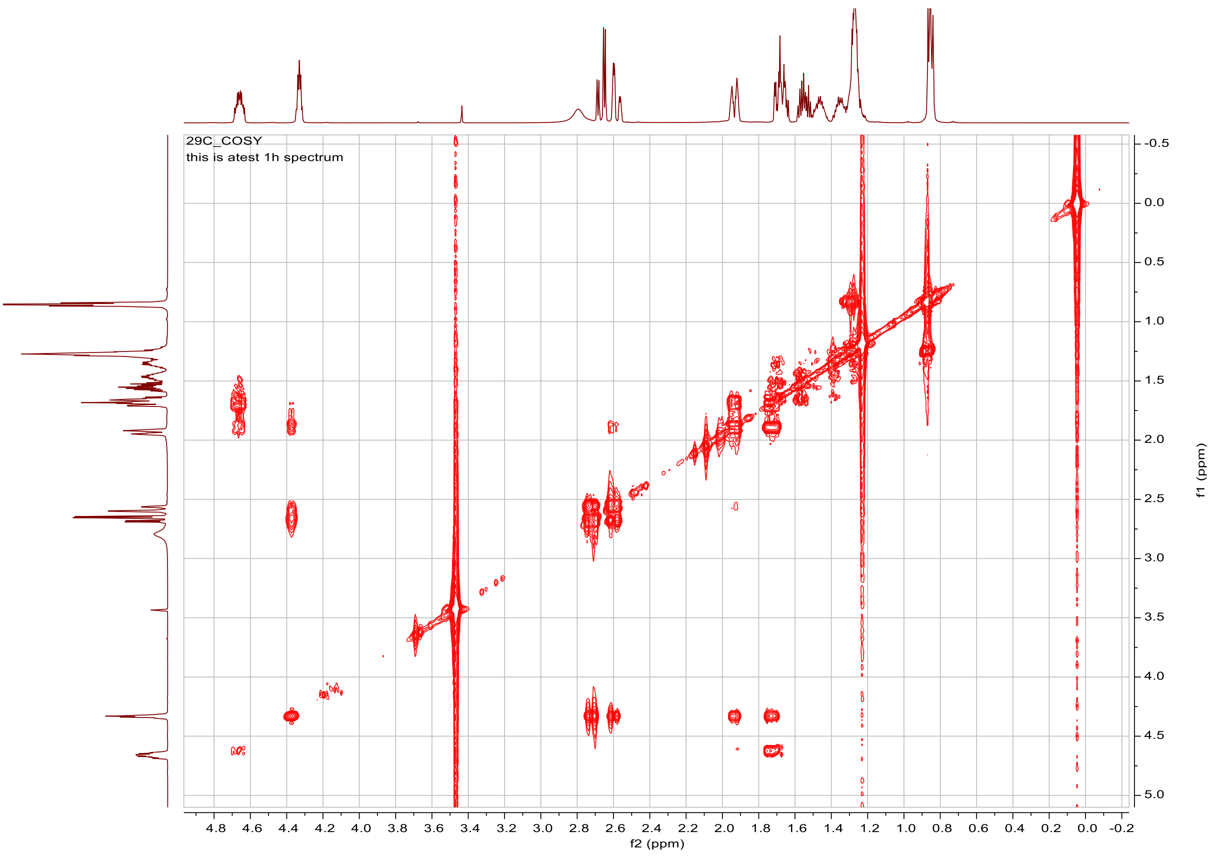

Figure 3. ^{1}H-^{1}H COSY spectrum of compound **1** (500 MHz, $CDCl_3$)

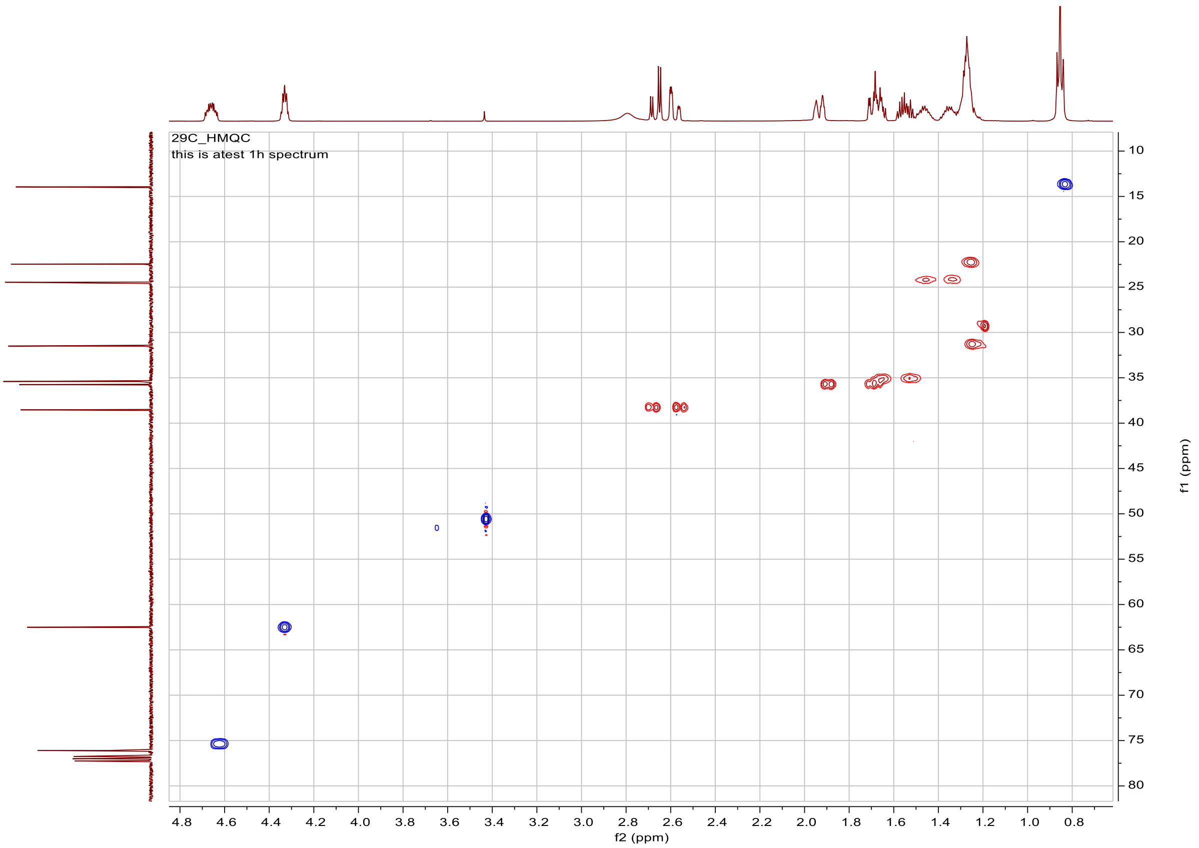

Figure 4. HMQC spectrum of compound **1** (500/125 MHz, $CDCl_3$)

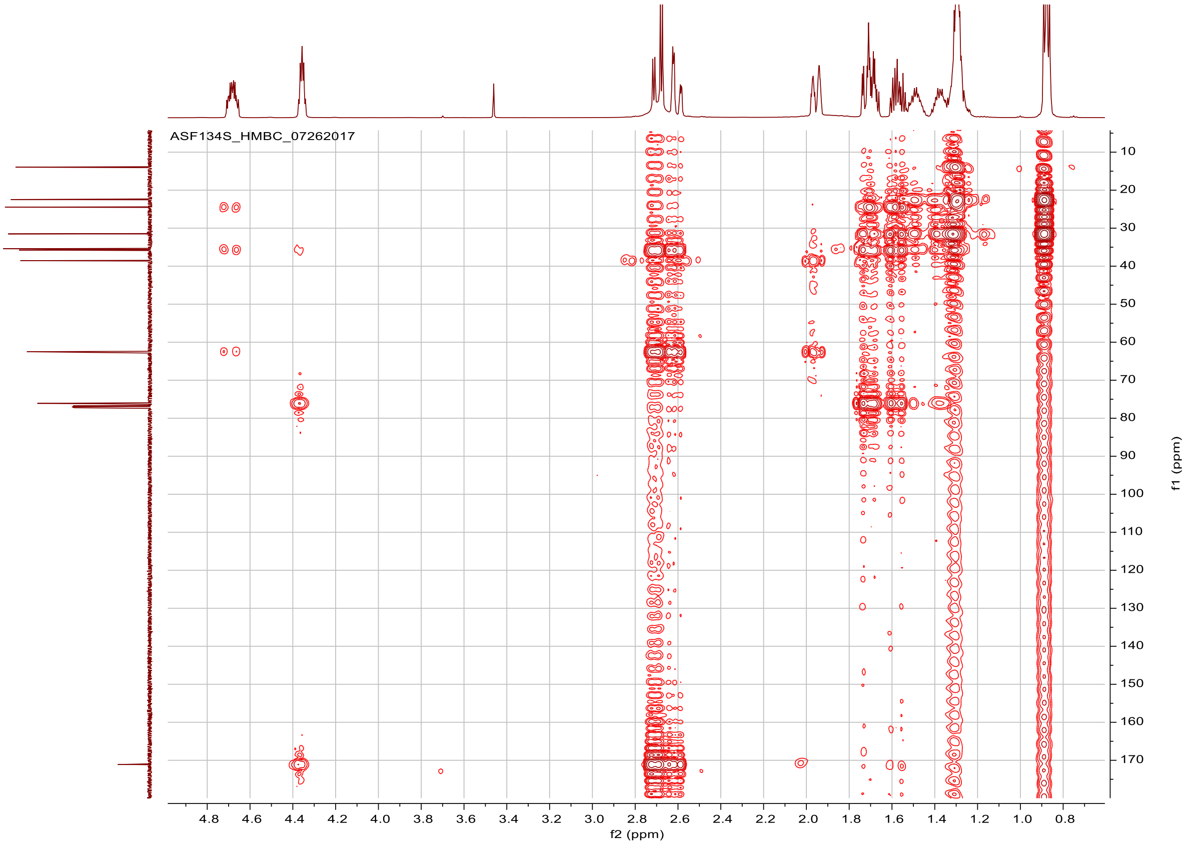

Figure 5. HMBC spectrum of compound **1** (500/125 MHz, $CDCl_3$)

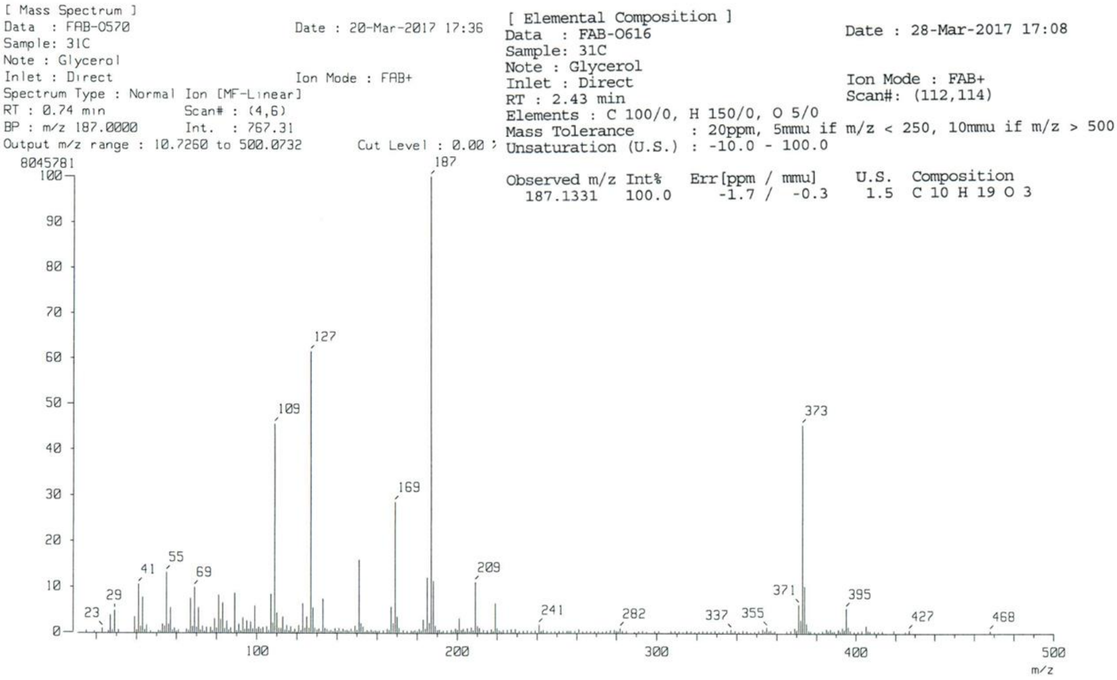

Figure 6. (+)HRFABMS spectrum and value of compound **1**

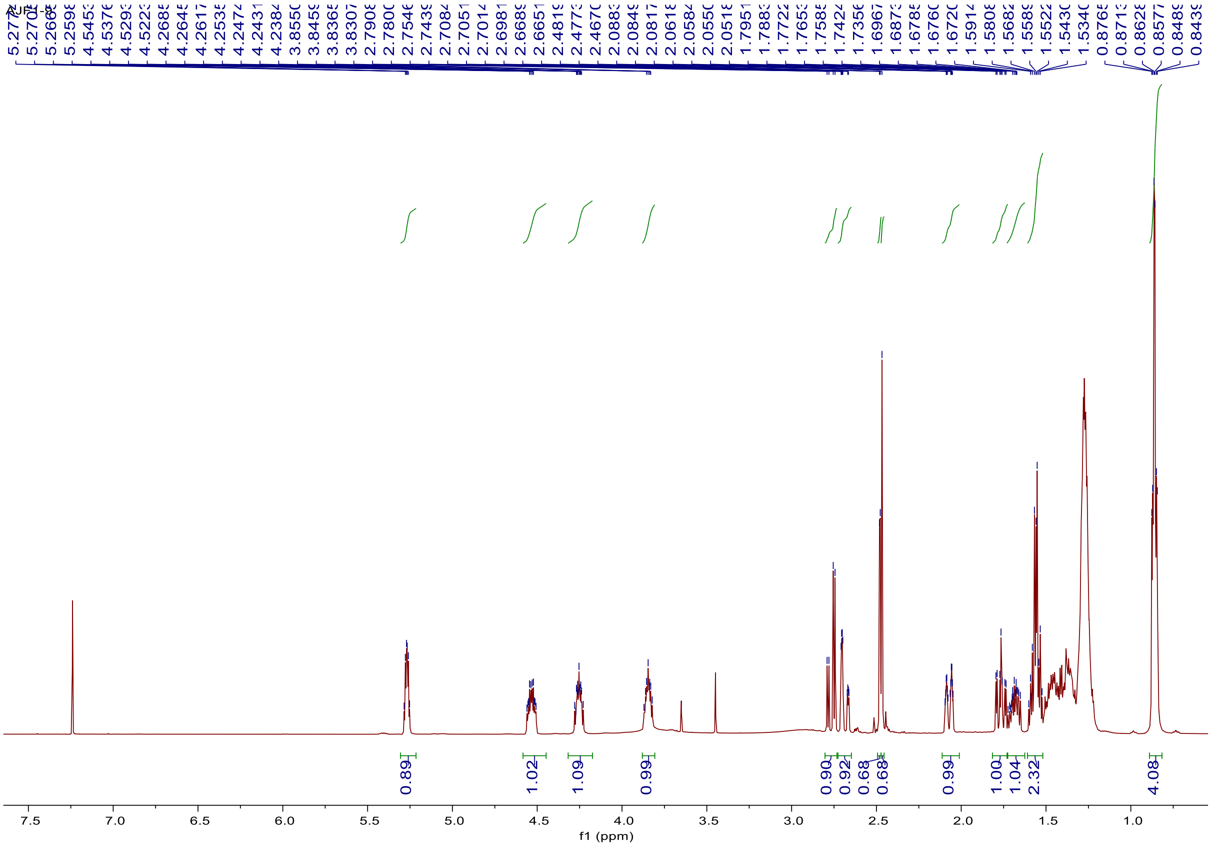

Figure 7. ^{1}H-NMR spectrum of compound **2** (500 MHz, $CDCl_3$)

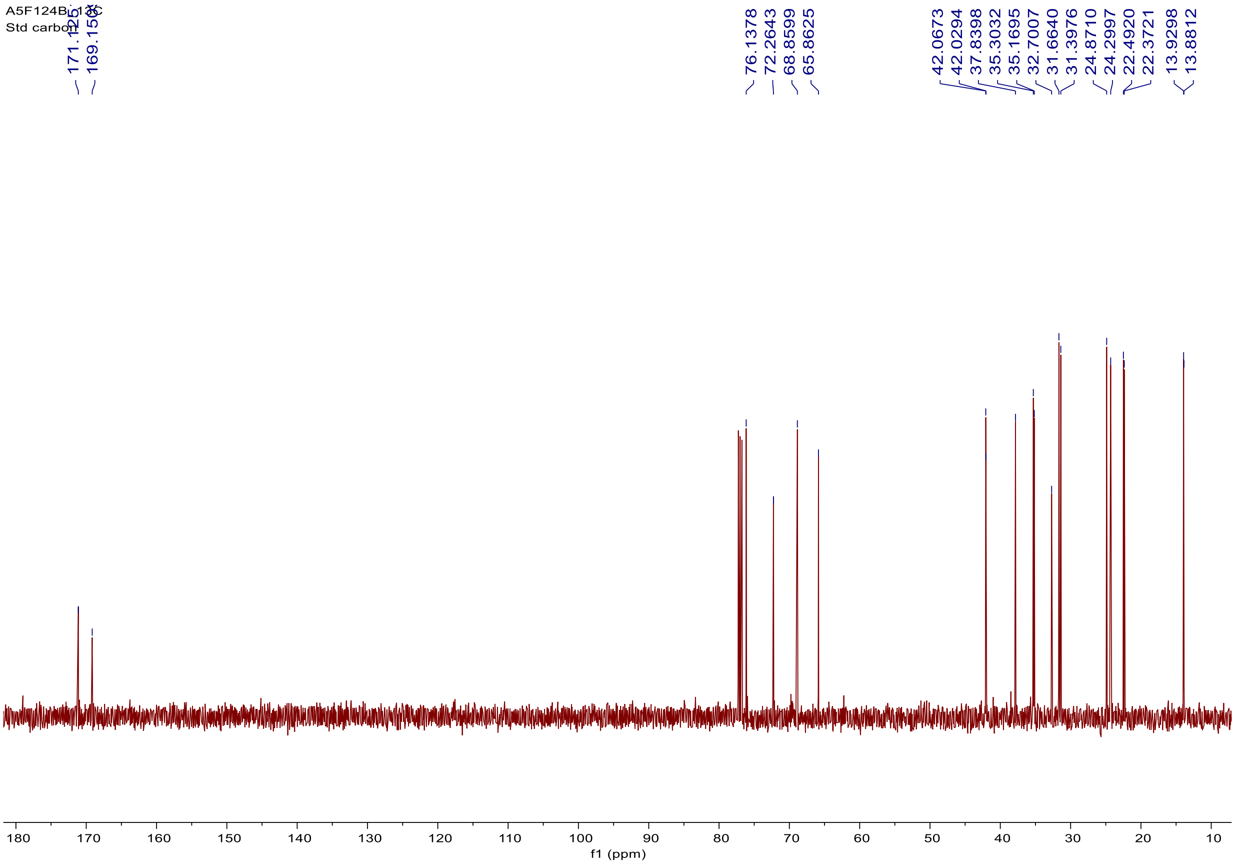

Figure 8. ^{13}C-NMR spectrum of compound **2** (125 MHz, $CDCl_3$)

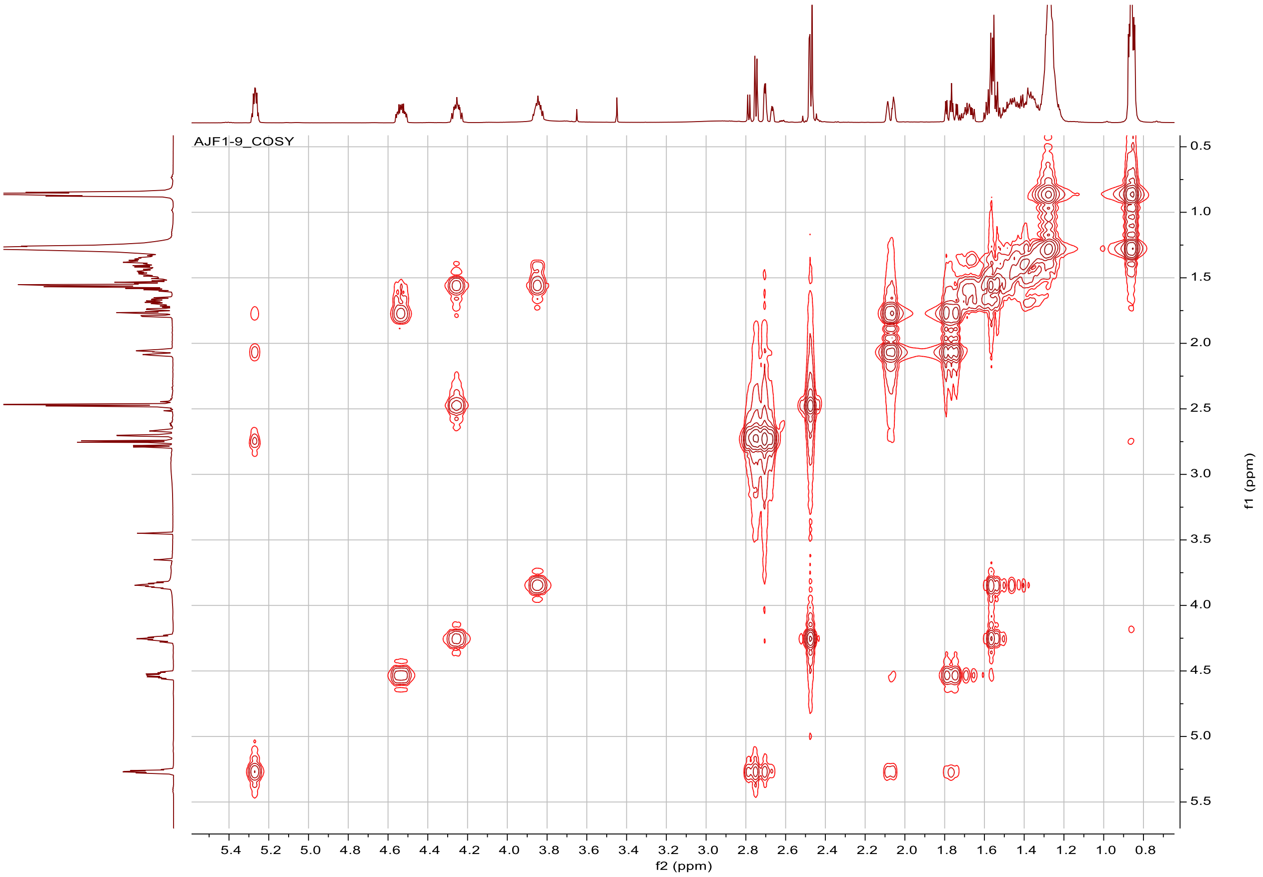

Figure 9. ^{1}H-^{1}H COSY spectrum of compound **2** (500 MHz, $CDCl_3$)

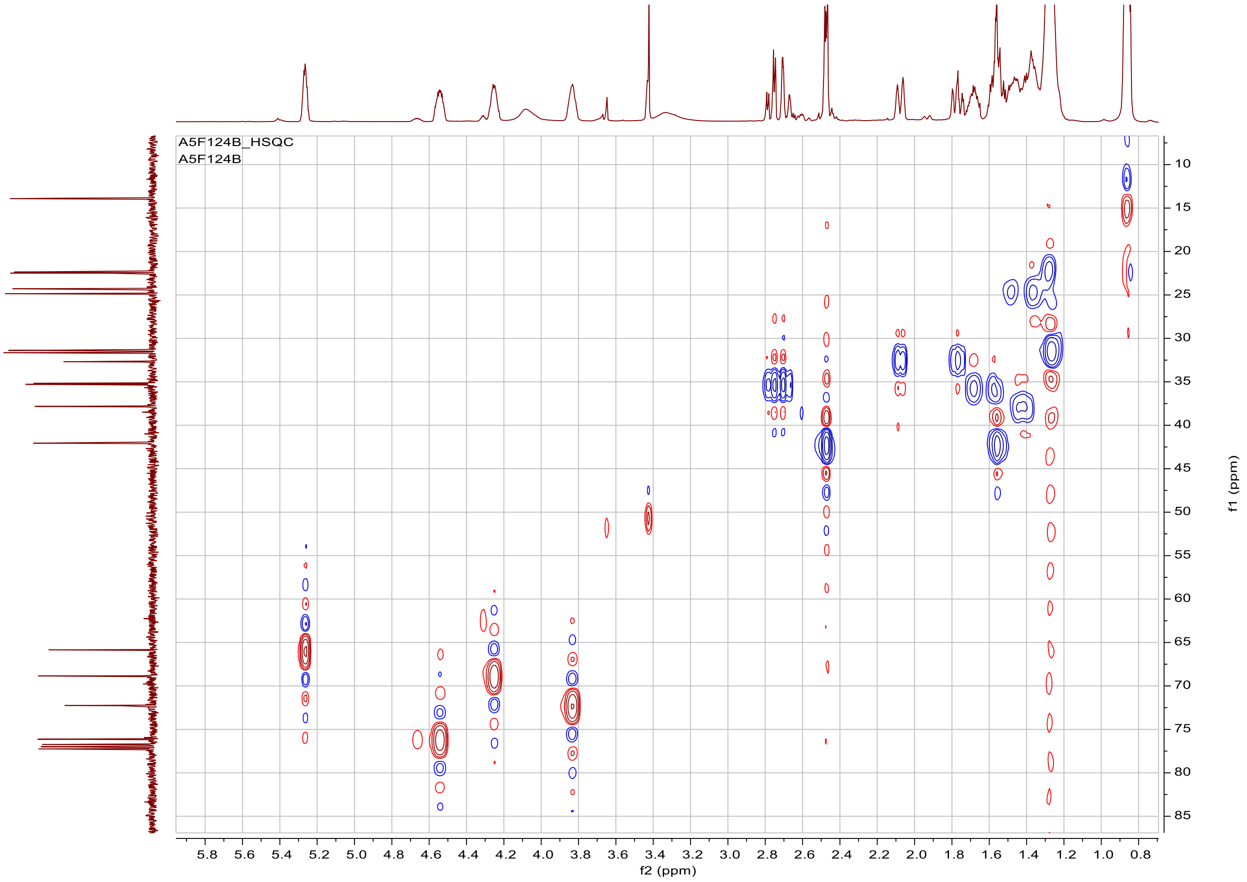

Figure 10. HMQC spectrum of compound **2** (500/125 MHz, $CDCl_3$)

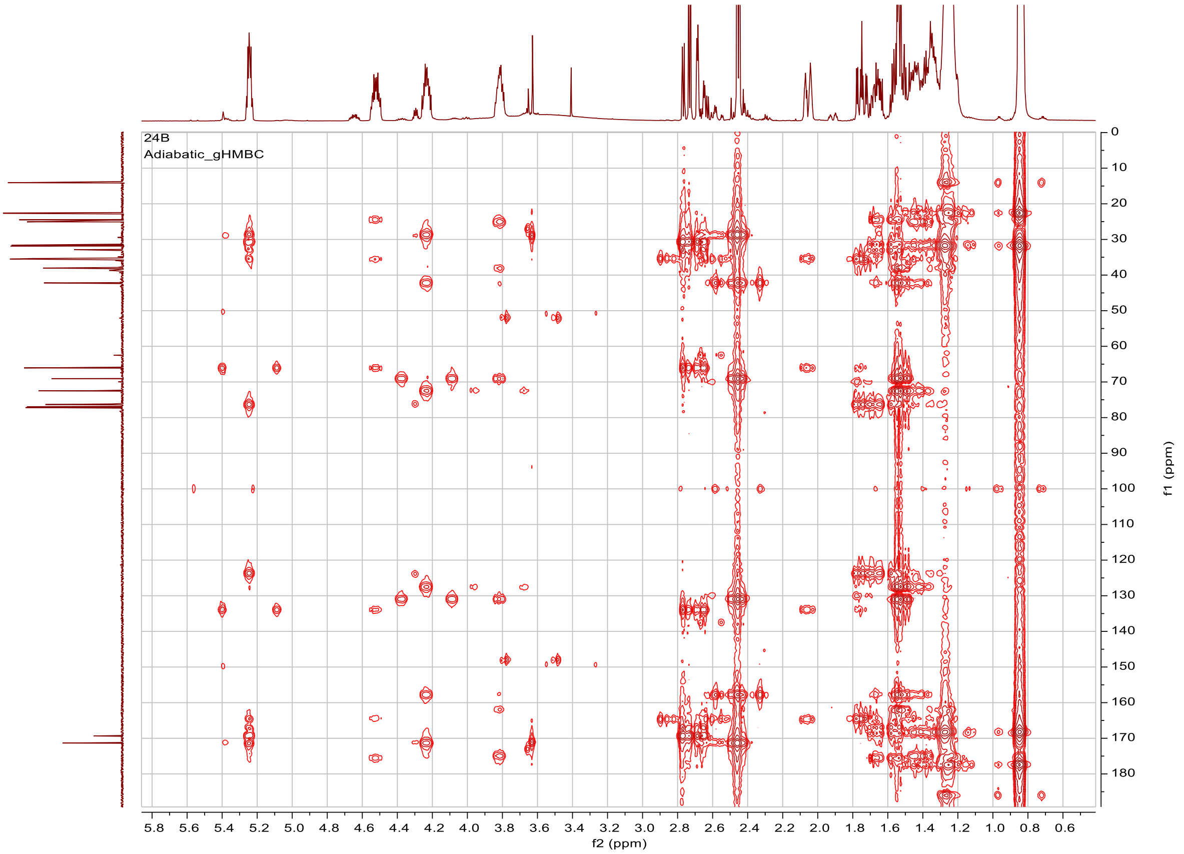

Figure 11a. HMBC spectrum of compound **2** (500/125 MHz, $CDCl_3$)

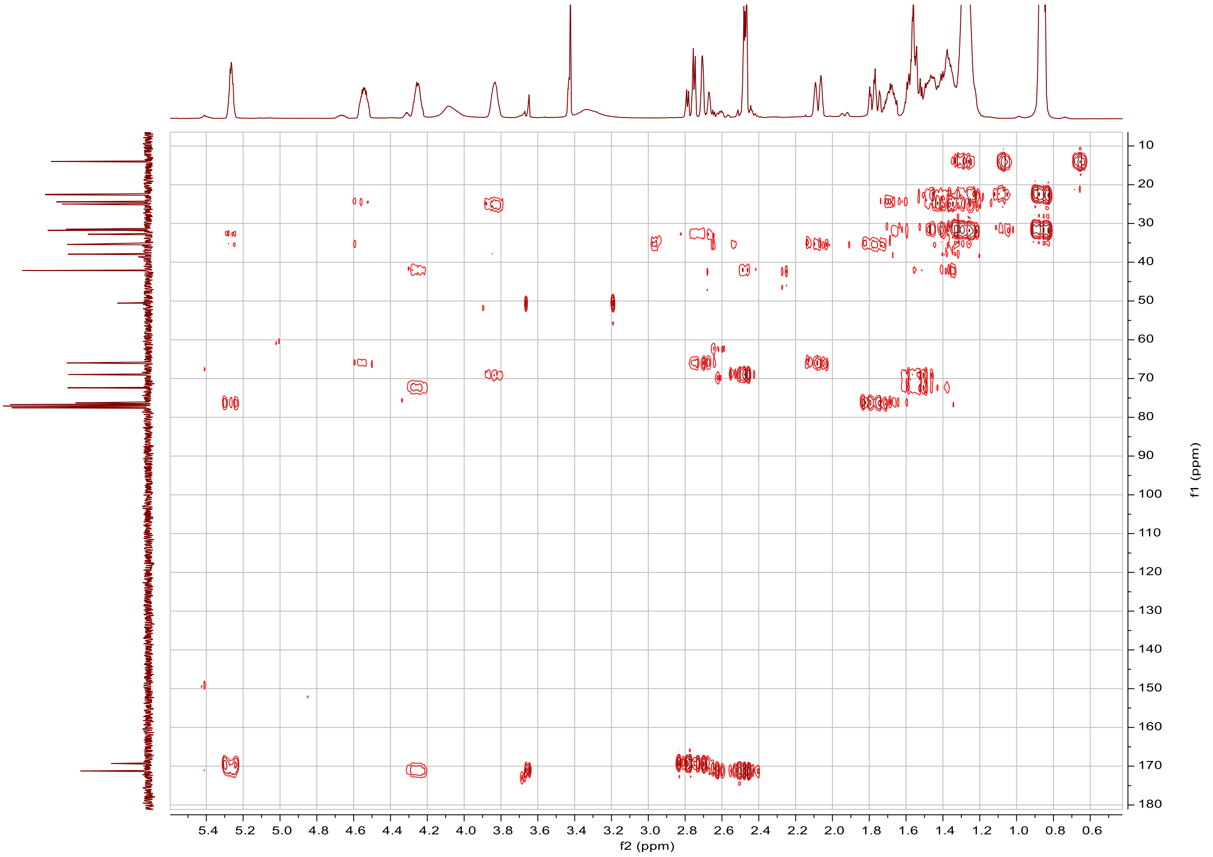

Figure 11b. HMBC spectrum of compound **2** (300/75 MHz, $CDCl_3$)

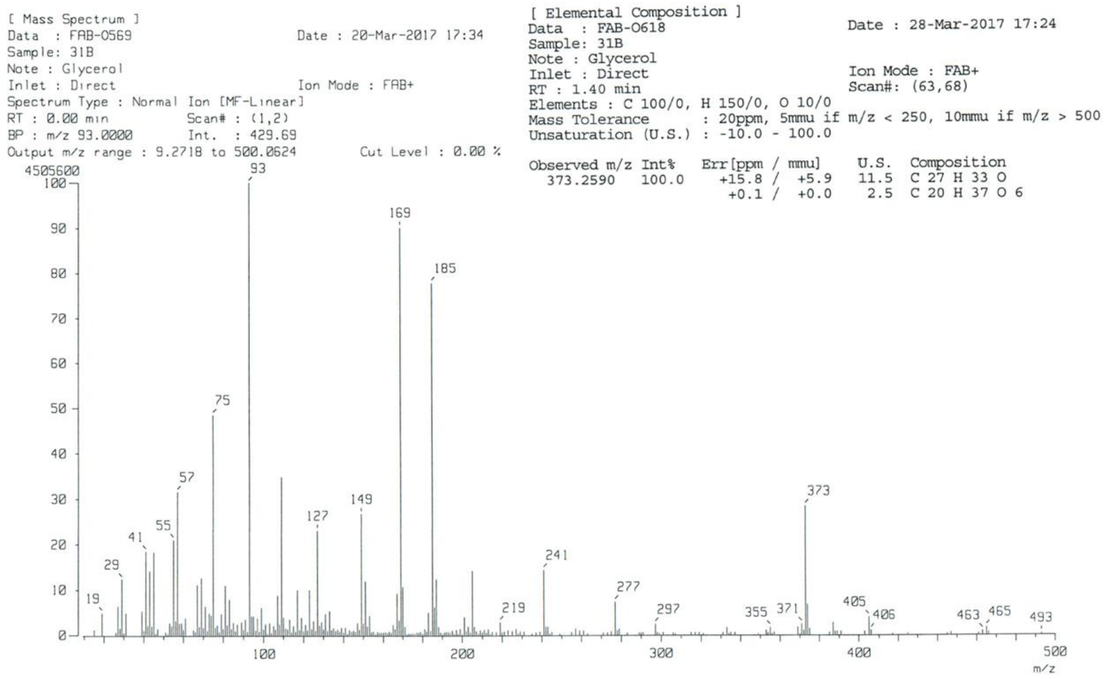

Figure 12. (+)HRFABMS spectrum and value of compound **2**

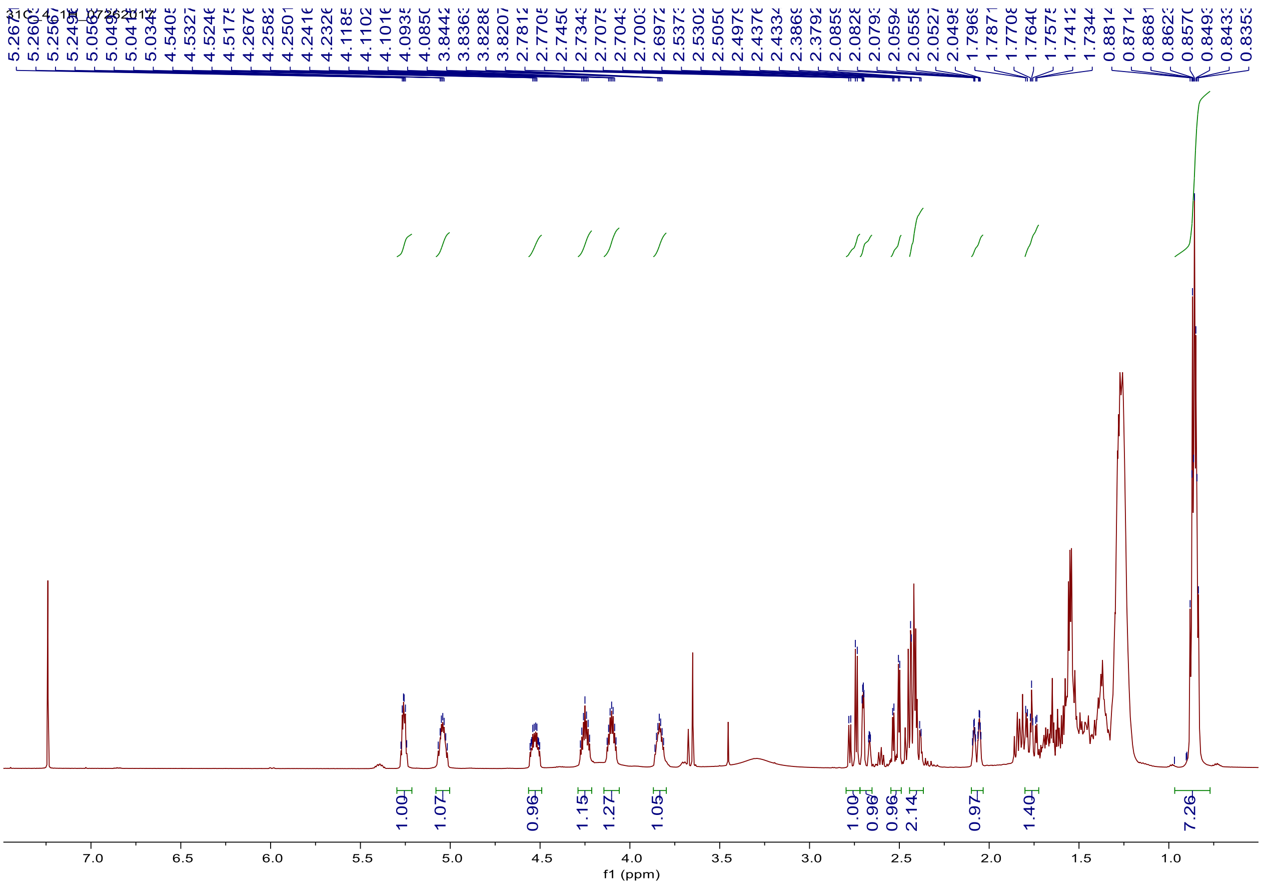

Figure 13. ^{1}H-NMR spectrum of compound **3** (500 MHz, $CDCl_3$)

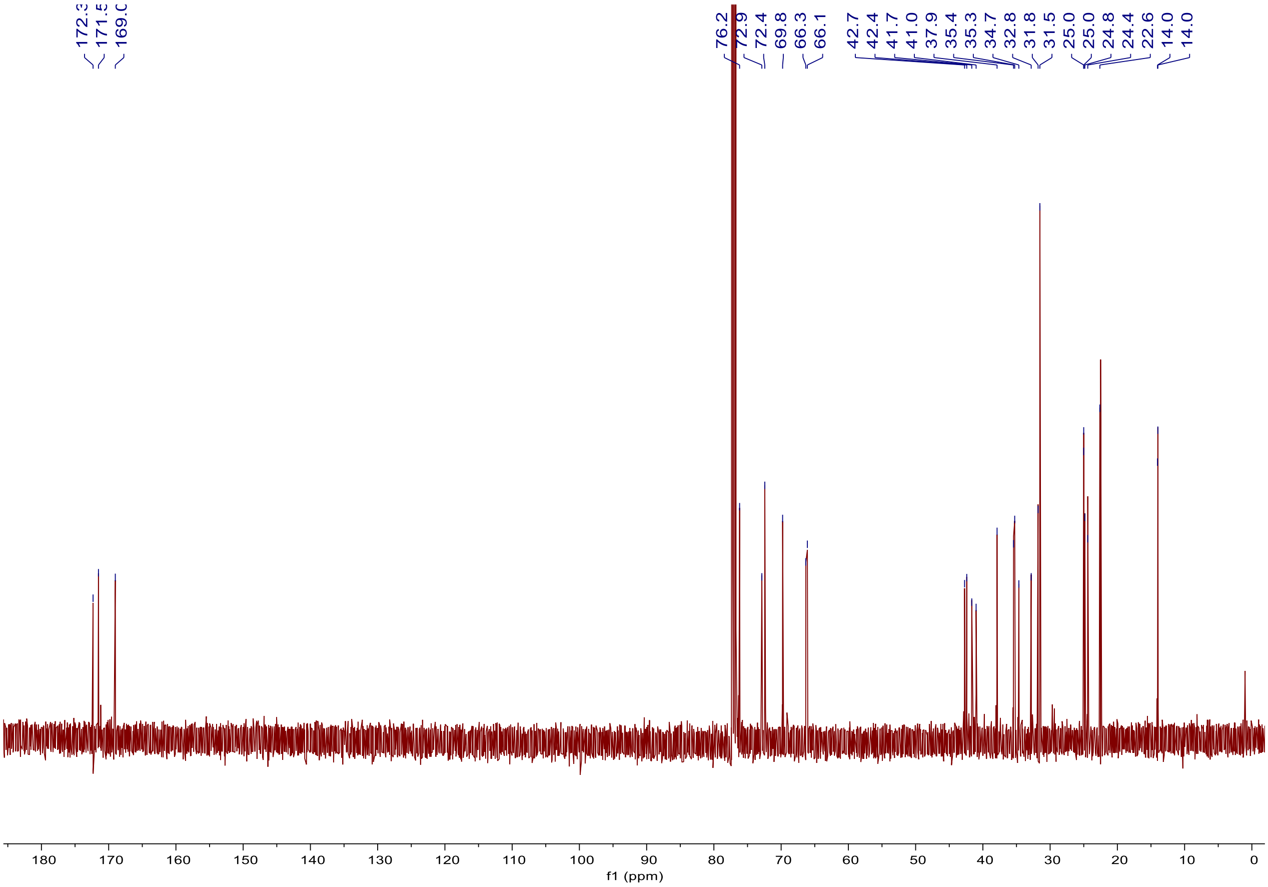

Figure 14. ^{13}C-NMR spectrum of compound **3** (125 MHz, $CDCl_3$)

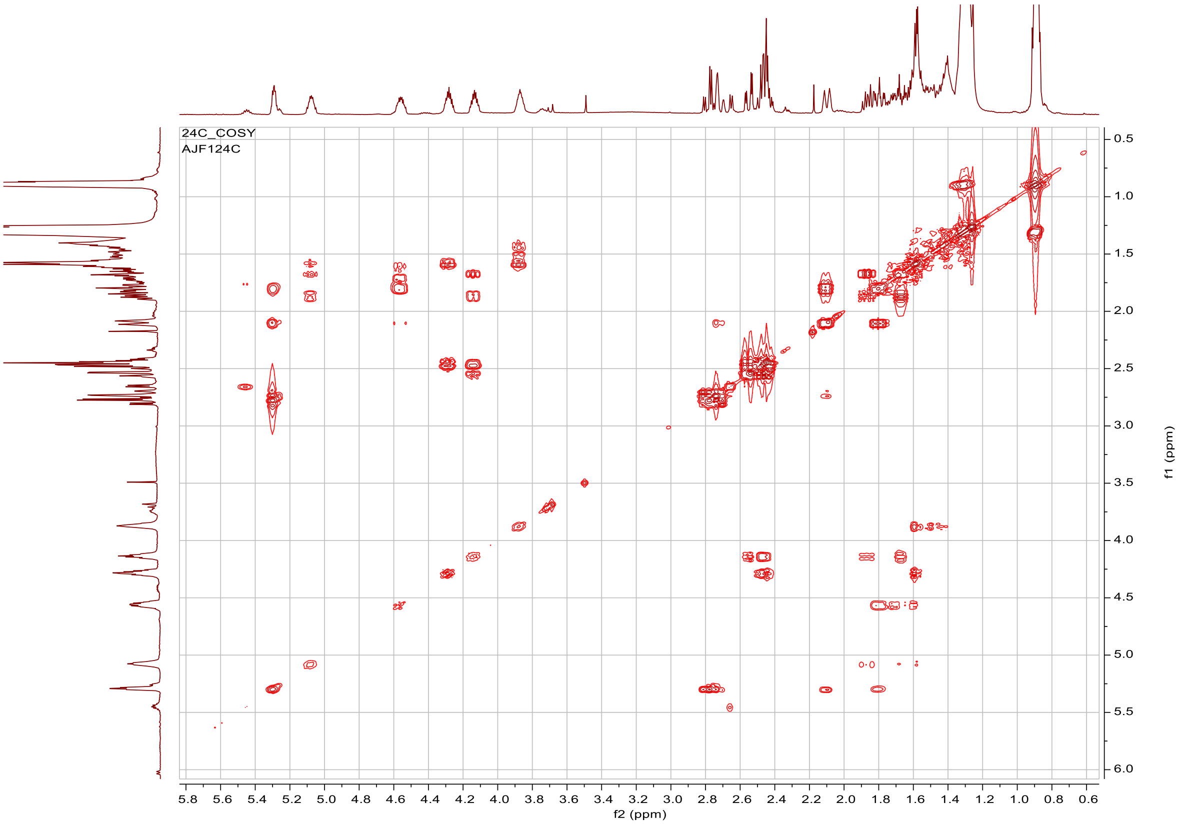

Figure 15. ^{1}H-^{1}H COSY spectrum of compound **3** (500 MHz, $CDCl_3$)

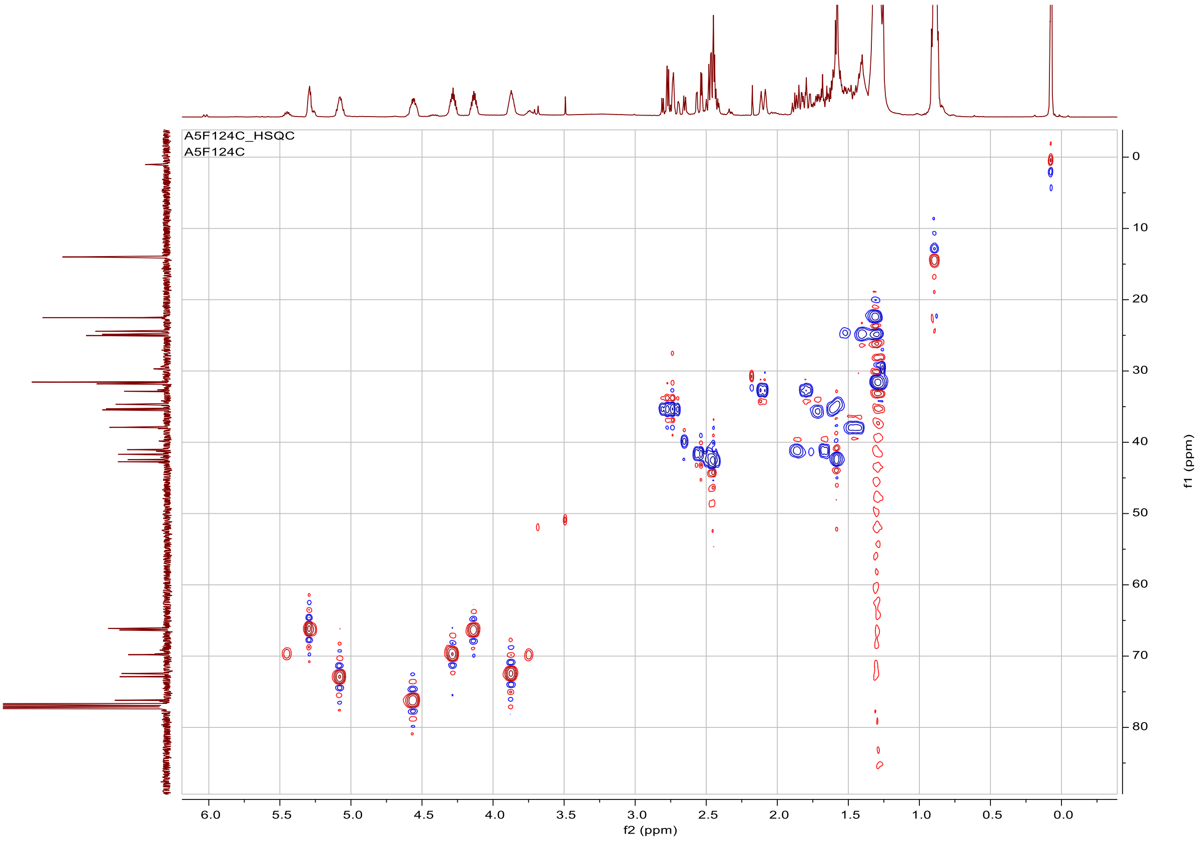

Figure 16. HMQC spectrum of compound **3** (500/125 MHz, $CDCl_3$)

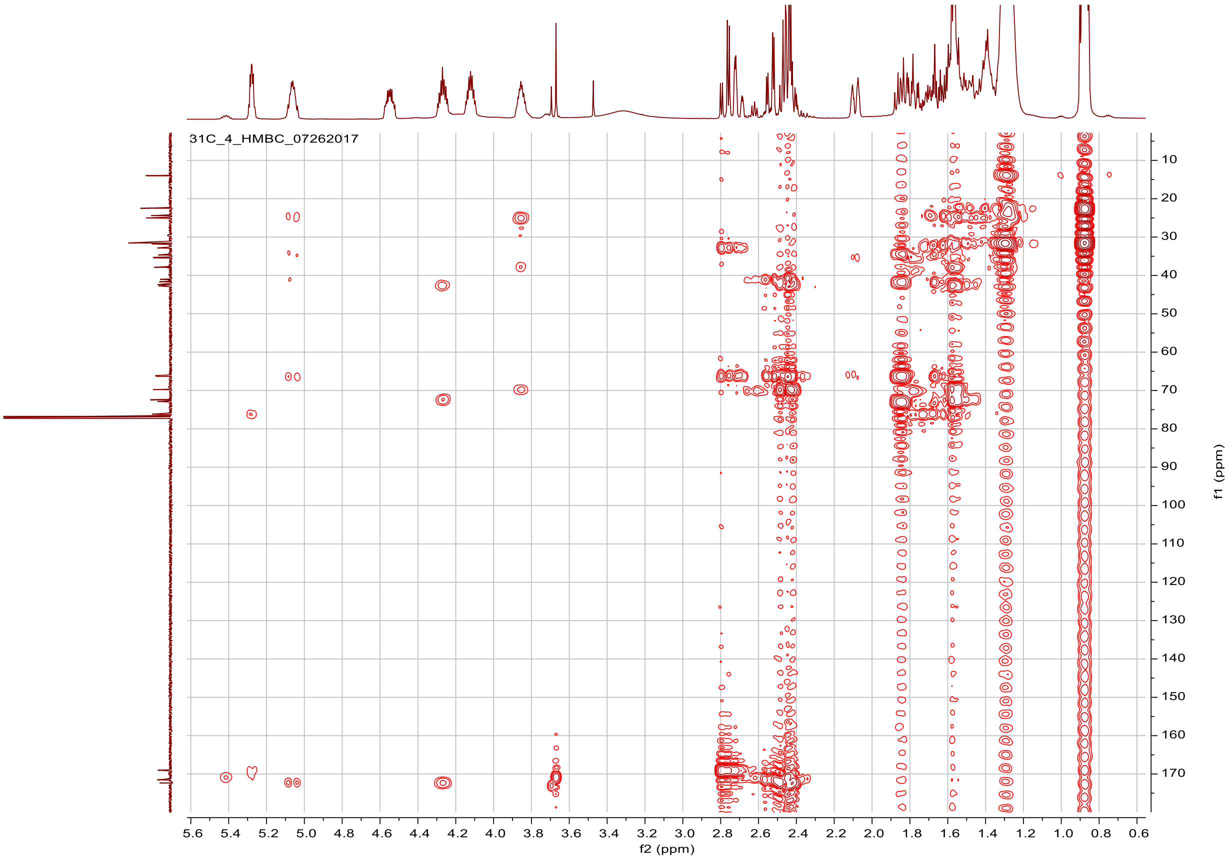

Figure 17. HMBC spectrum of compound **3** (500/125 MHz, $CDCl_3$)

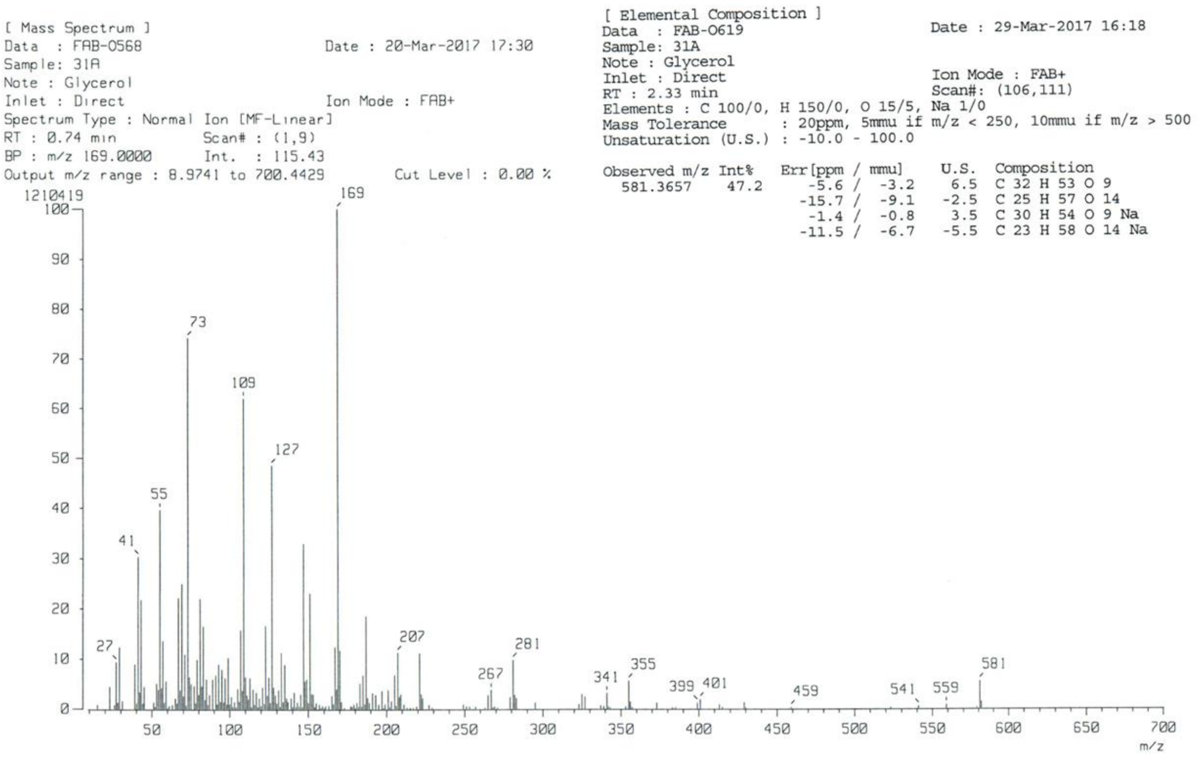

Figure 18. (+)HRFABMS spectrum and value of compound **3**

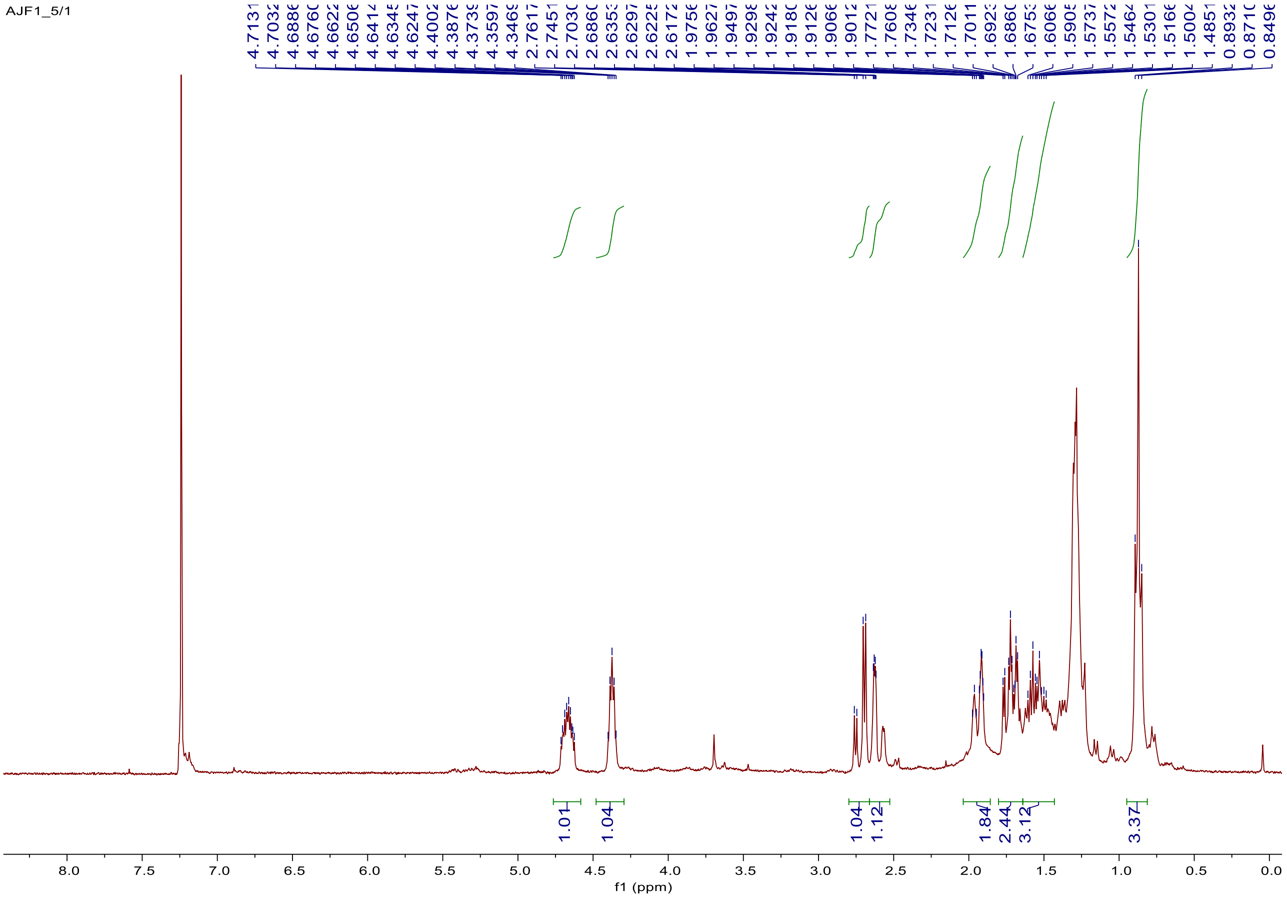

Figure 19. ^{1}H-NMR spectrum of compound **2a** (300 MHz, $CDCl_3$)

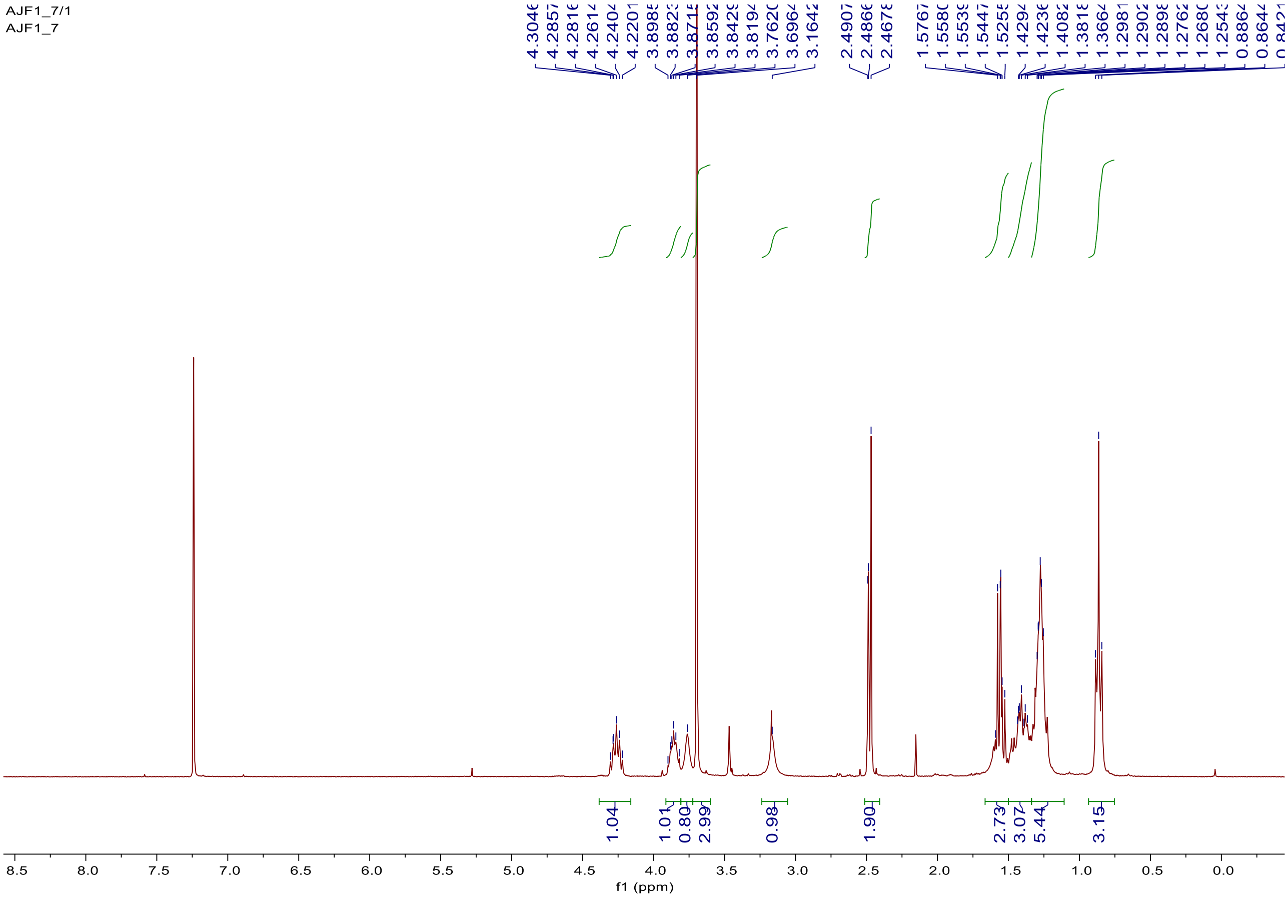

Figure 20. ^{1}H-NMR spectrum of compound **2b** (300 MHz, $CDCl_3$)

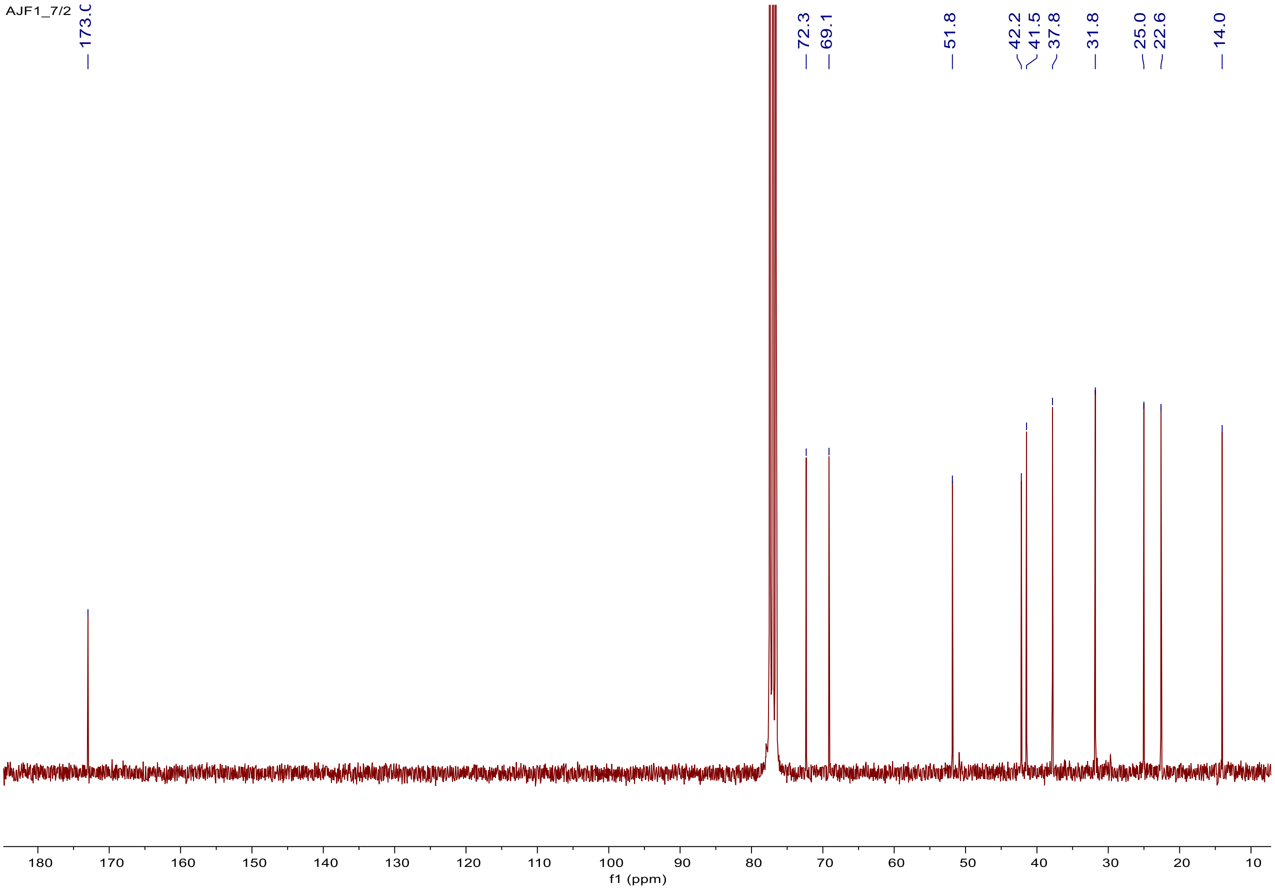

Figure 21. ^{13}C-NMR spectrum of compound **2b** (75 MHz, $CDCl_3$)

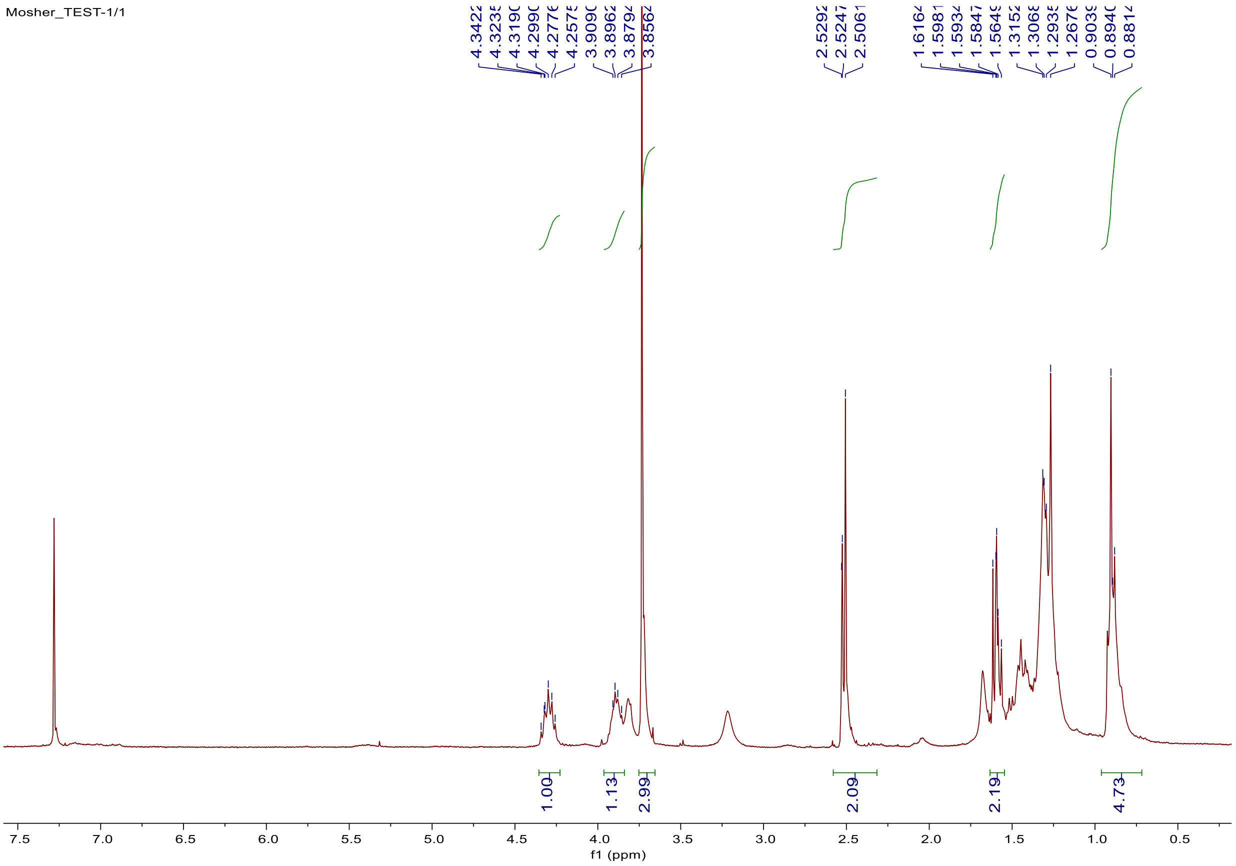

Figure 22. ^{1}H-NMR spectrum of compound **3d** (300 MHz, $CDCl_3$)

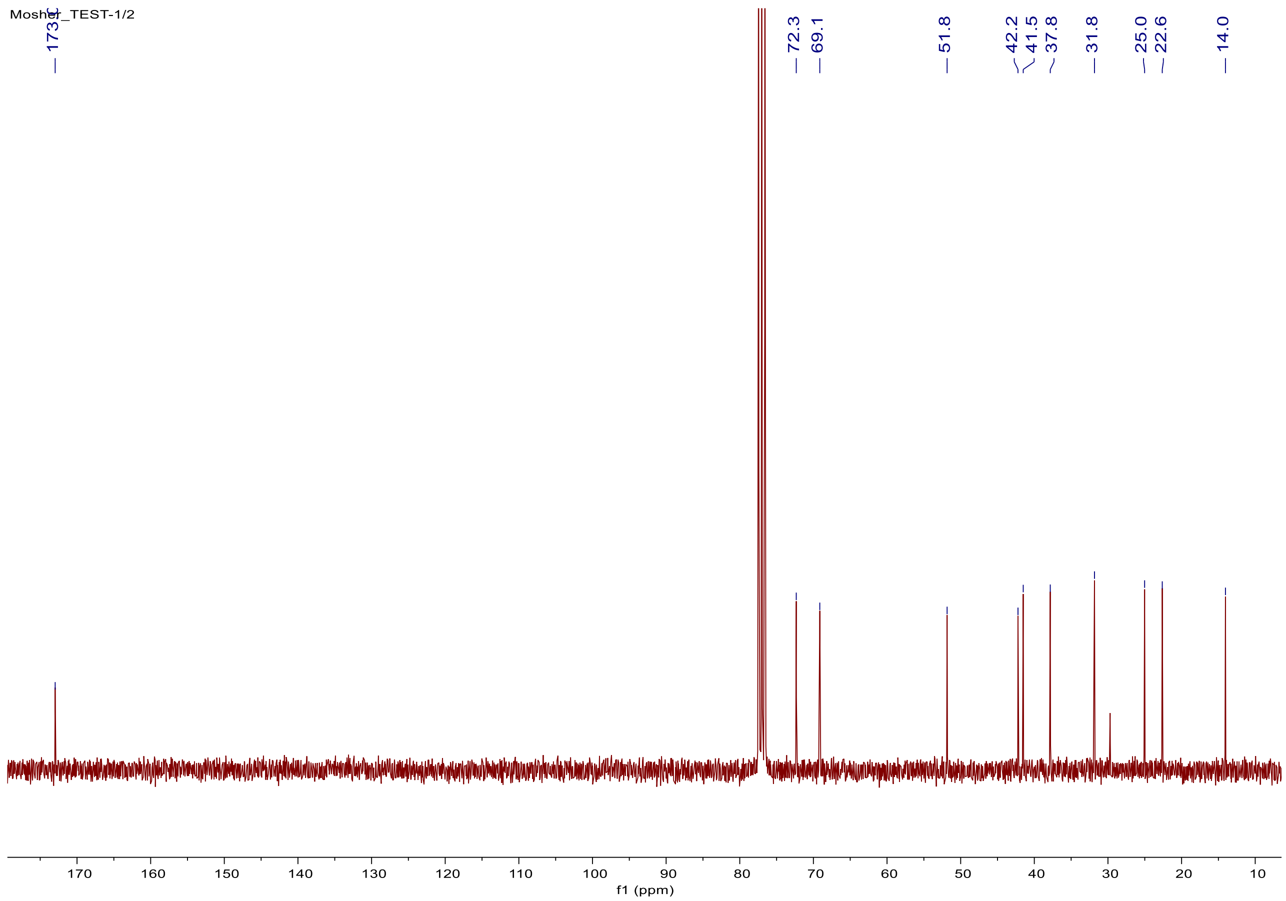

Figure 23. ^{13}C-NMR spectrum of compound **3d** (75 MHz, $CDCl_3$)

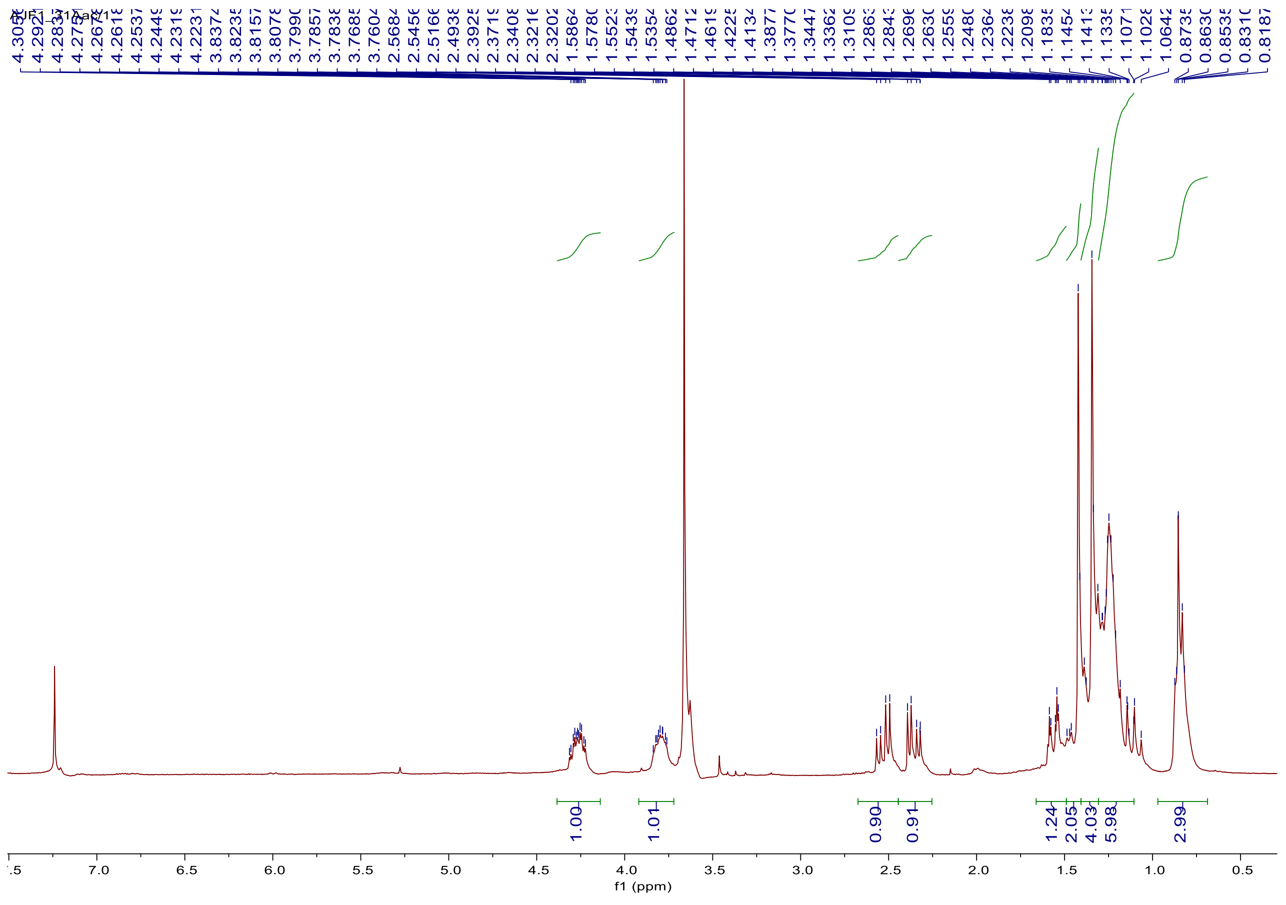

Figure 24. ^{1}H-NMR spectrum of compound **1a** (300 MHz, $CDCl_3$)

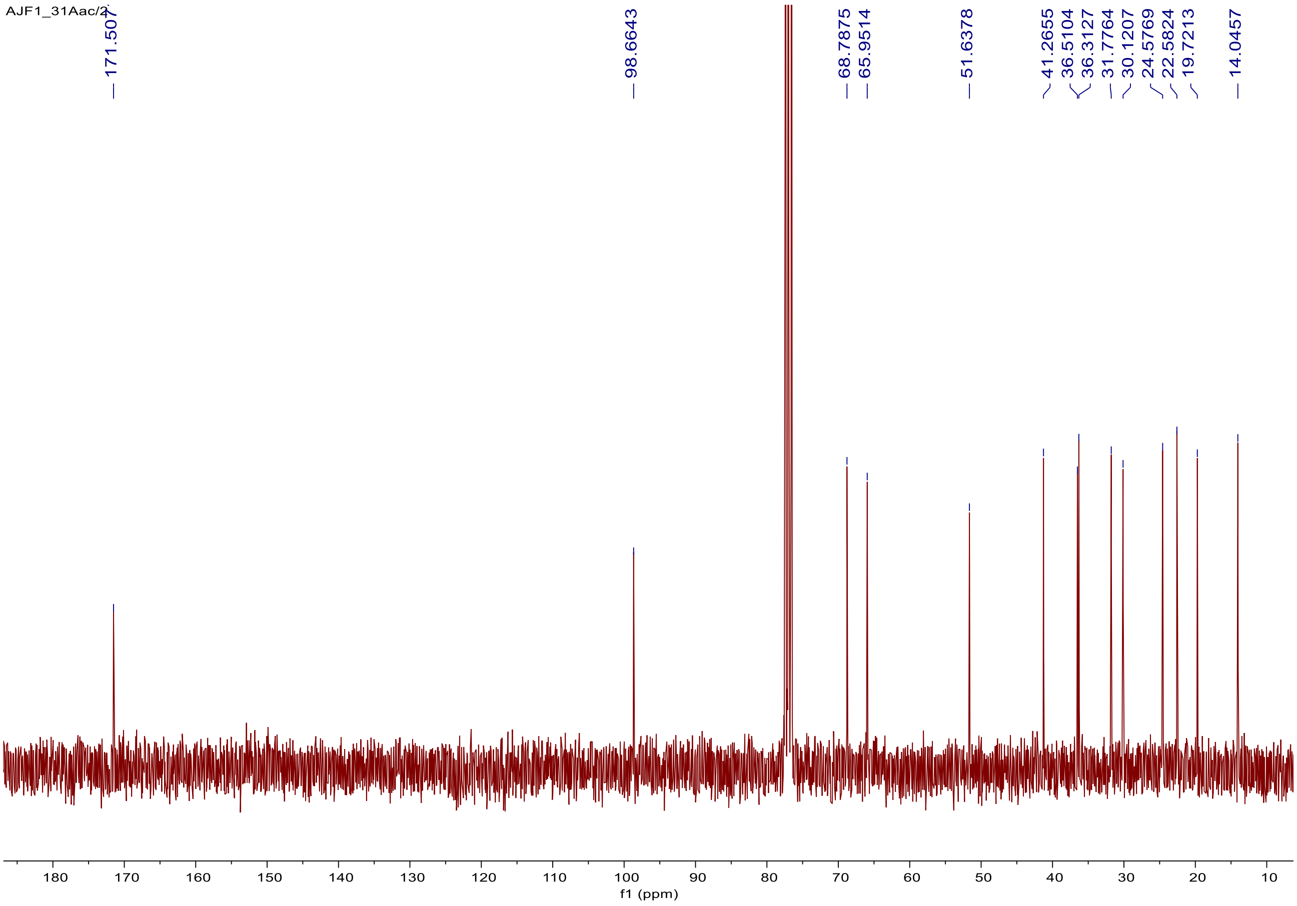

Figure 25. ^{13}C-NMR spectrum of compound **1a** (75 MHz, $CDCl_3$)

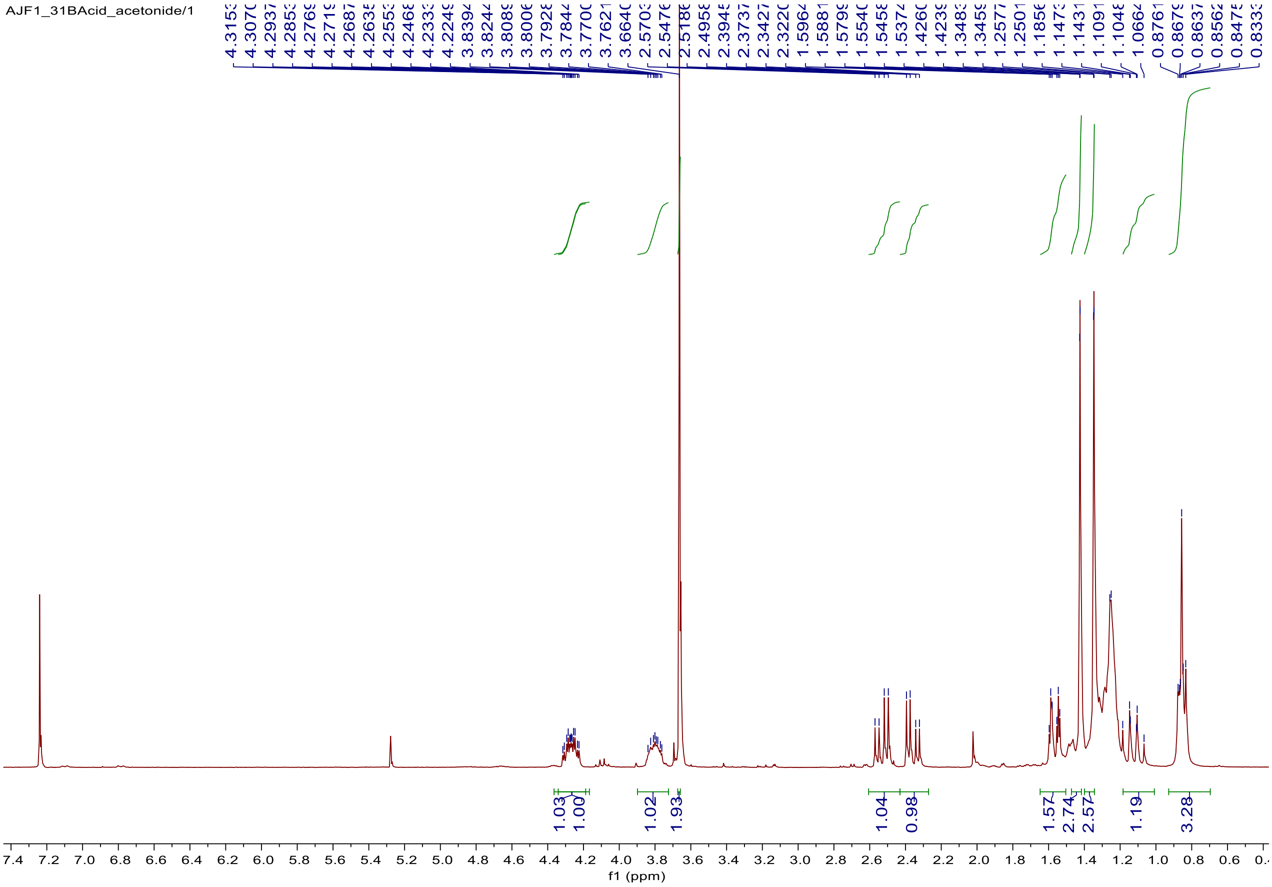

Figure 26. ^{1}H-NMR spectrum of compound **2c** (300 MHz, $CDCl_3$)

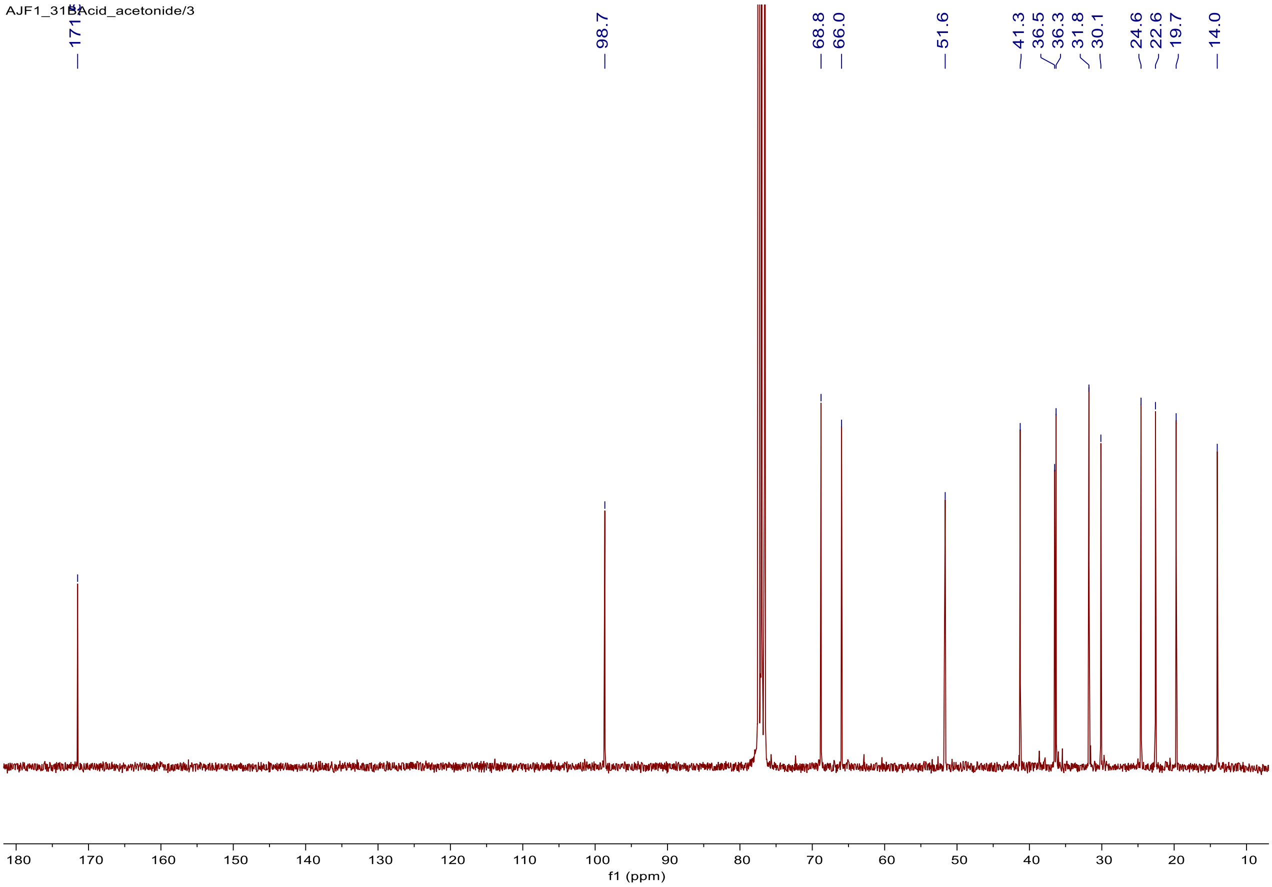

Figure 27. ^{13}C-NMR spectrum of compound **2c** (75 MHz, $CDCl_3$)

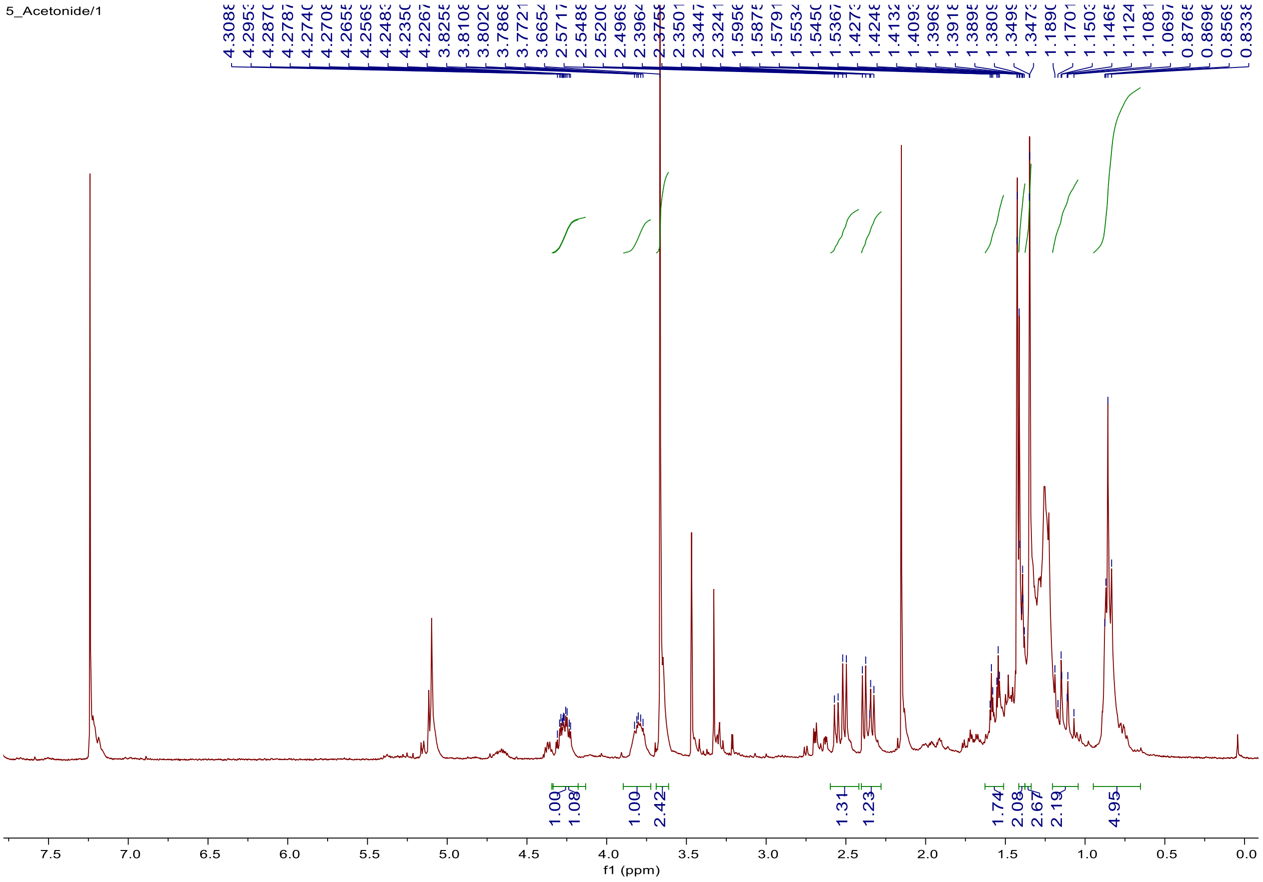

Figure 28. ^{1}H-NMR spectrum of compound **2d** (300 MHz, $CDCl_3$)

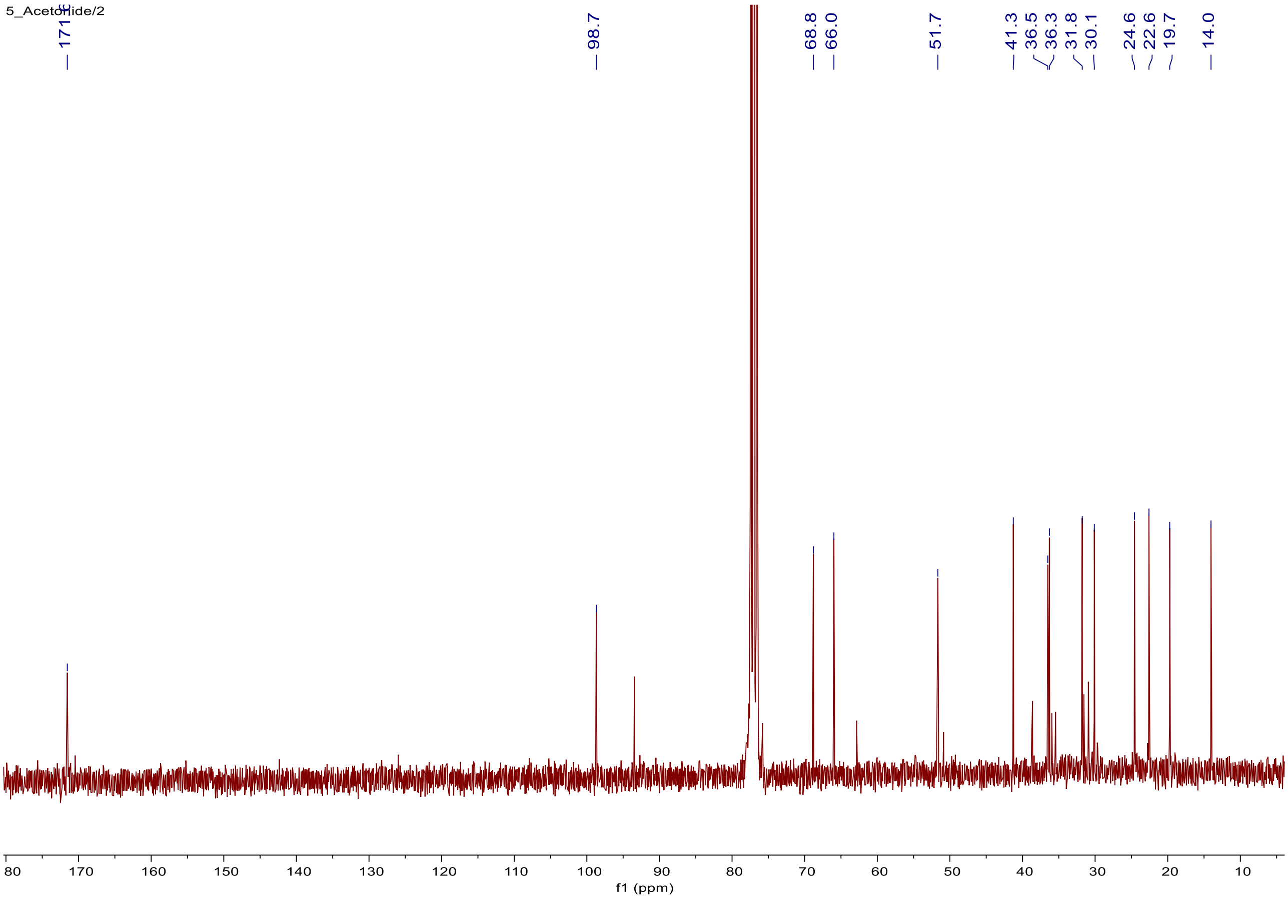

Figure 29. ^{13}C-NMR spectrum of compound **2d** (75 MHz, $CDCl_3$)

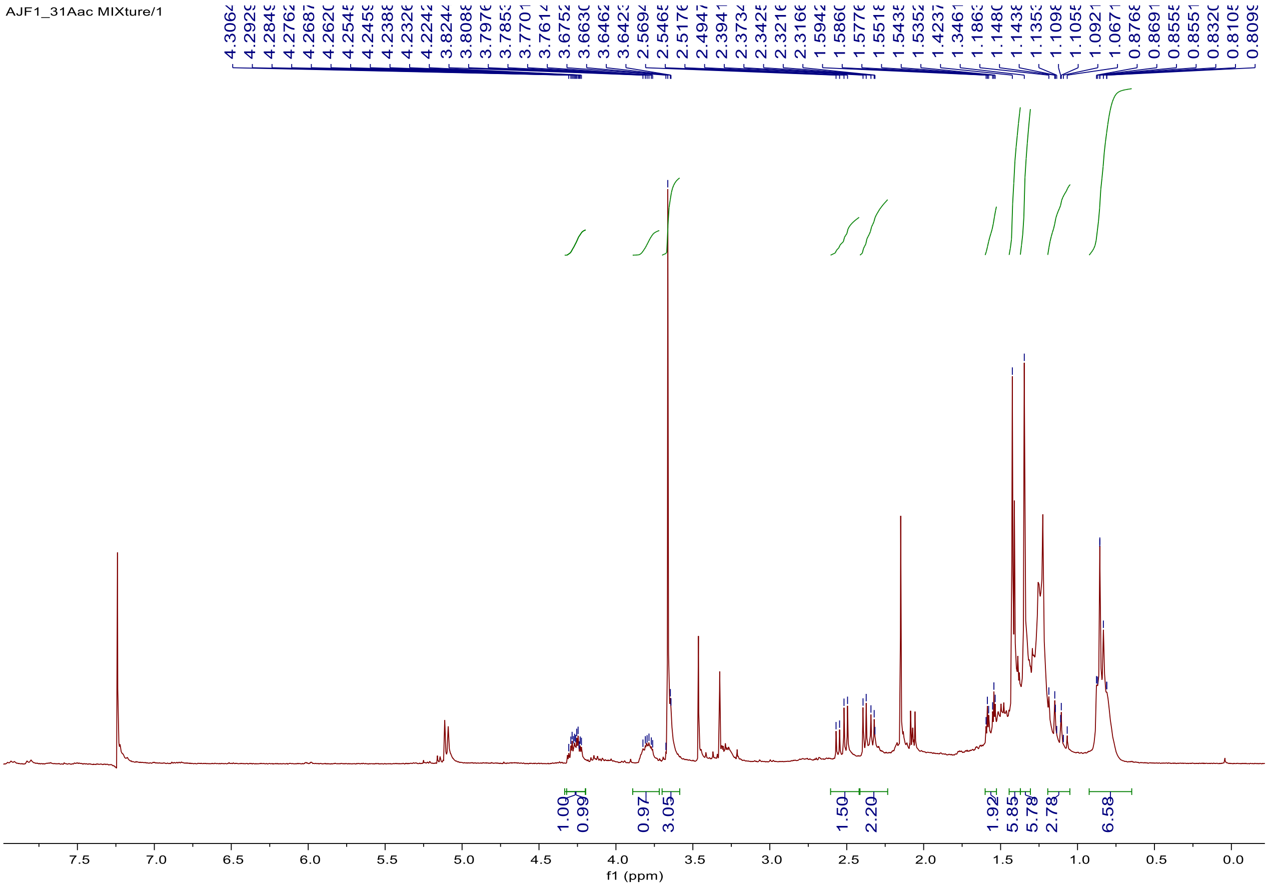

Figure 30. ^{1}H-NMR spectrum of compound **3c** (300 MHz, $CDCl_3$)

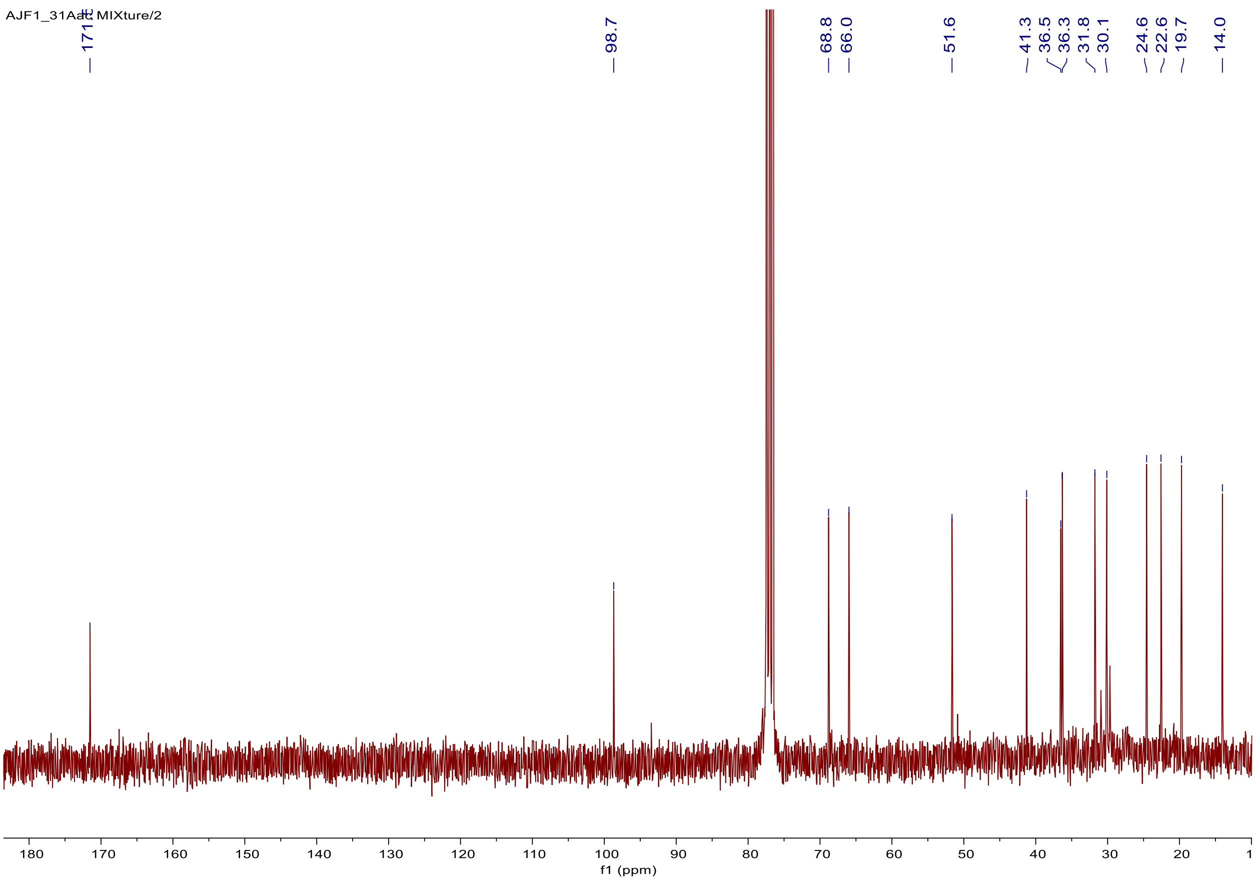

Figure 31. ^{13}C-NMR spectrum of compound **3c** (75 MHz, $CDCl_3$)

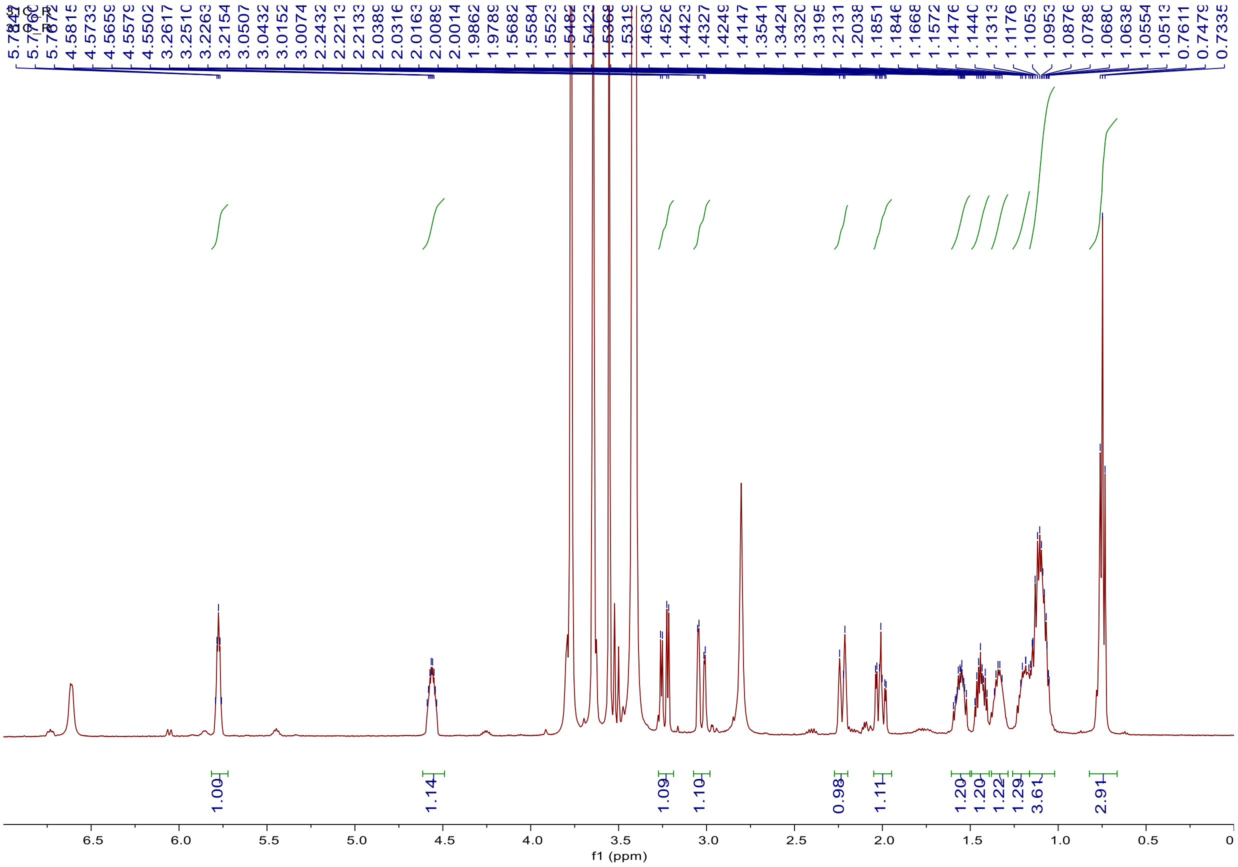

Figure 32. ^{1}H-NMR spectrum of (*S*)-MTPA ester of **1** (500 MHz, Pyridine-d_5)

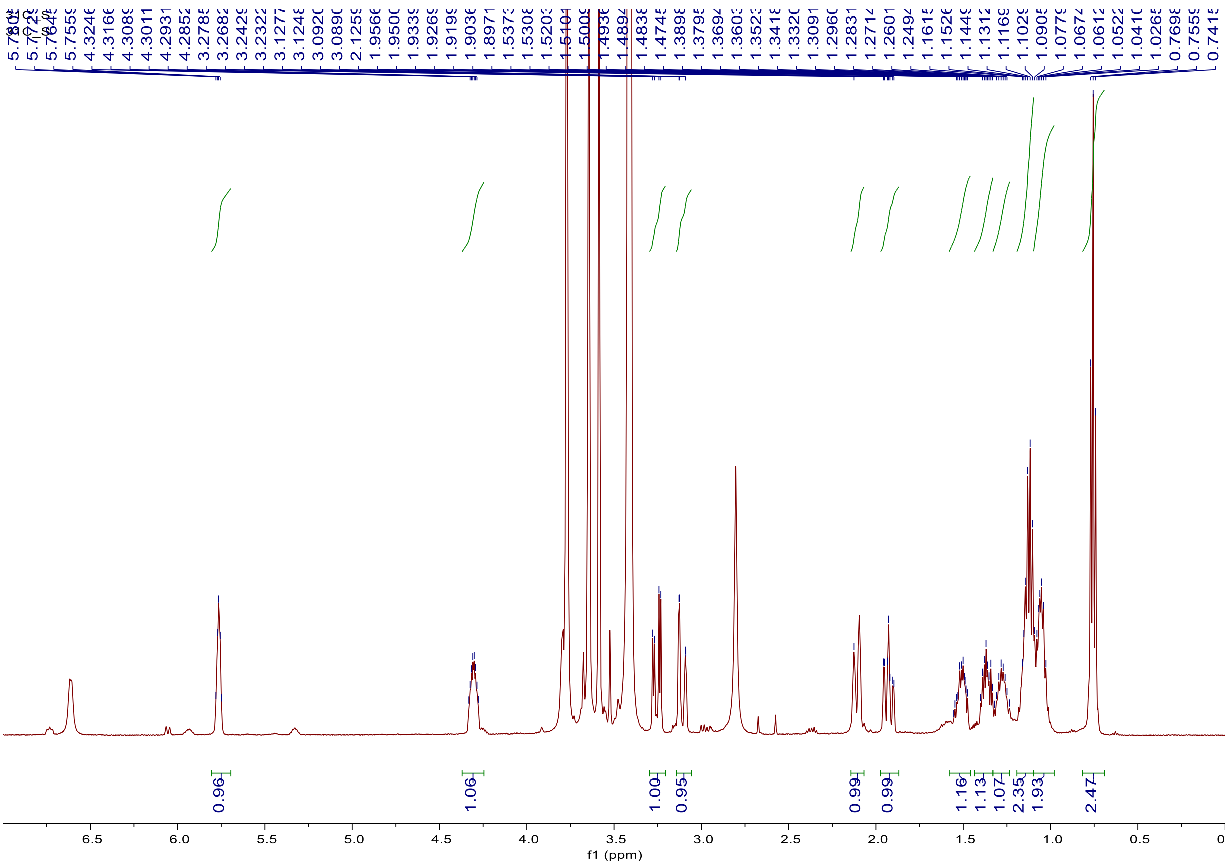

Figure 33. ^{1}H-NMR spectrum of (*R*)-MTPA ester of **1** (300 MHz, Pyridine-d_5)

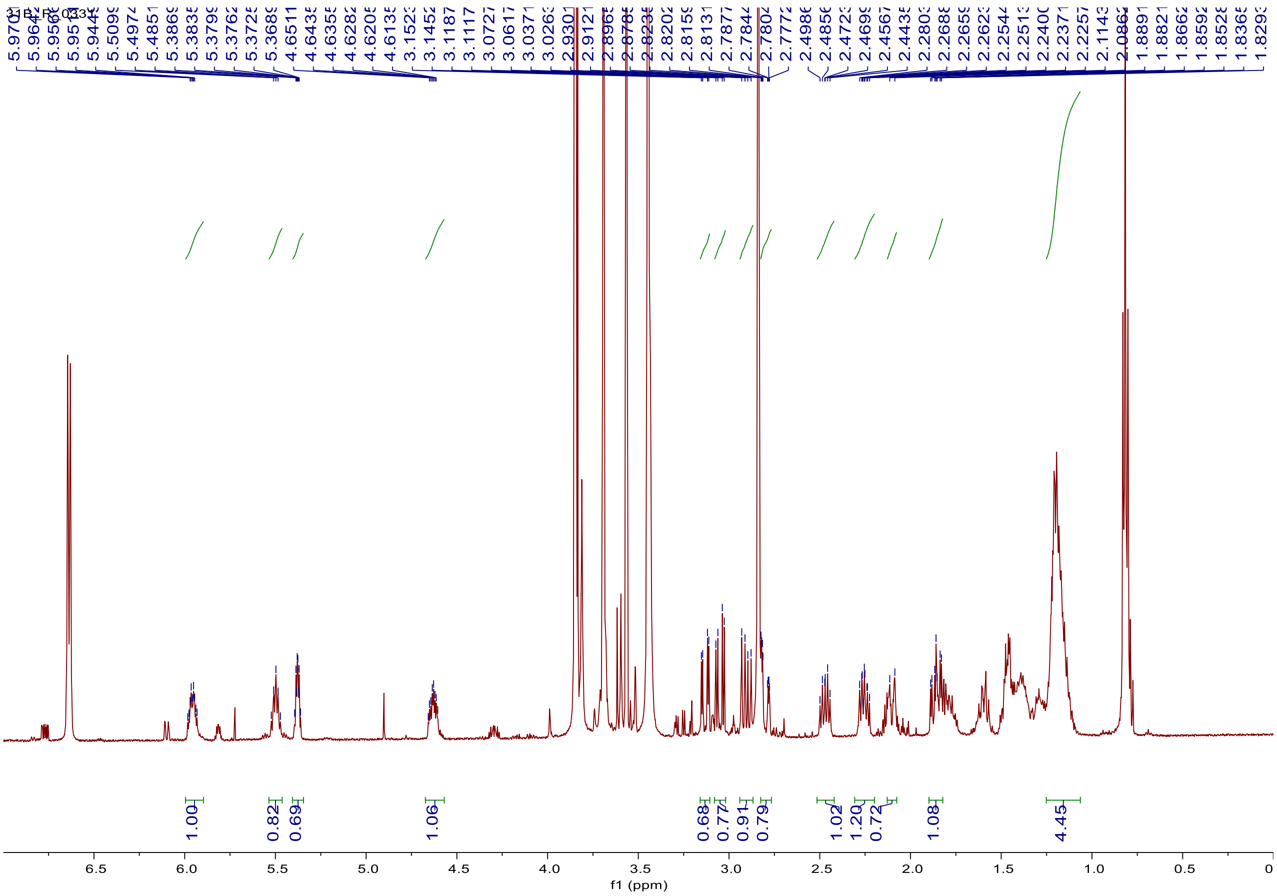

Figure 34. ^{1}H-NMR spectrum of (*S*)-MTPA ester of **2** (300 MHz, Pyridine-d_5)

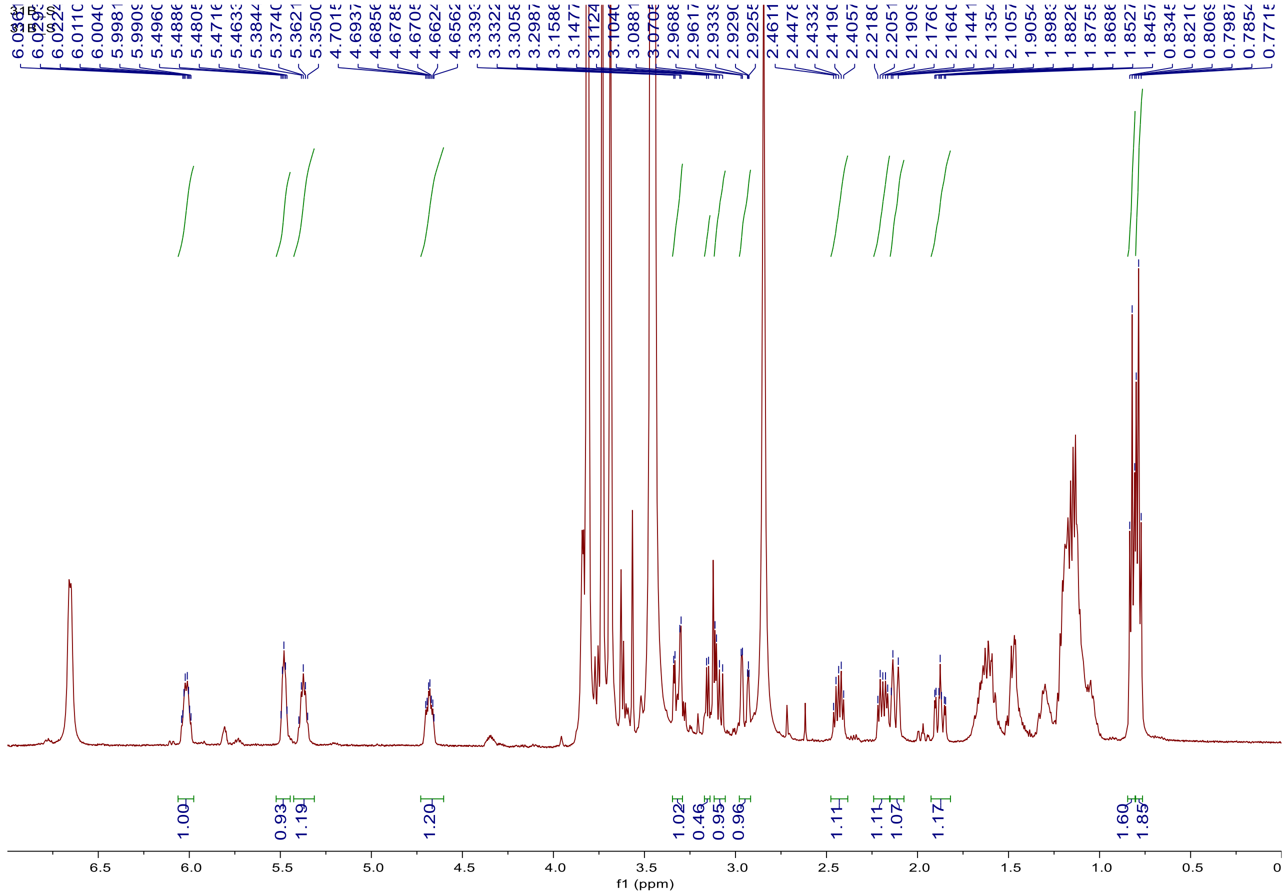

Figure 35. ^{1}H-NMR spectrum of (*R*)-MTPA ester of **2** (300 MHz, Pyridine-d_5)

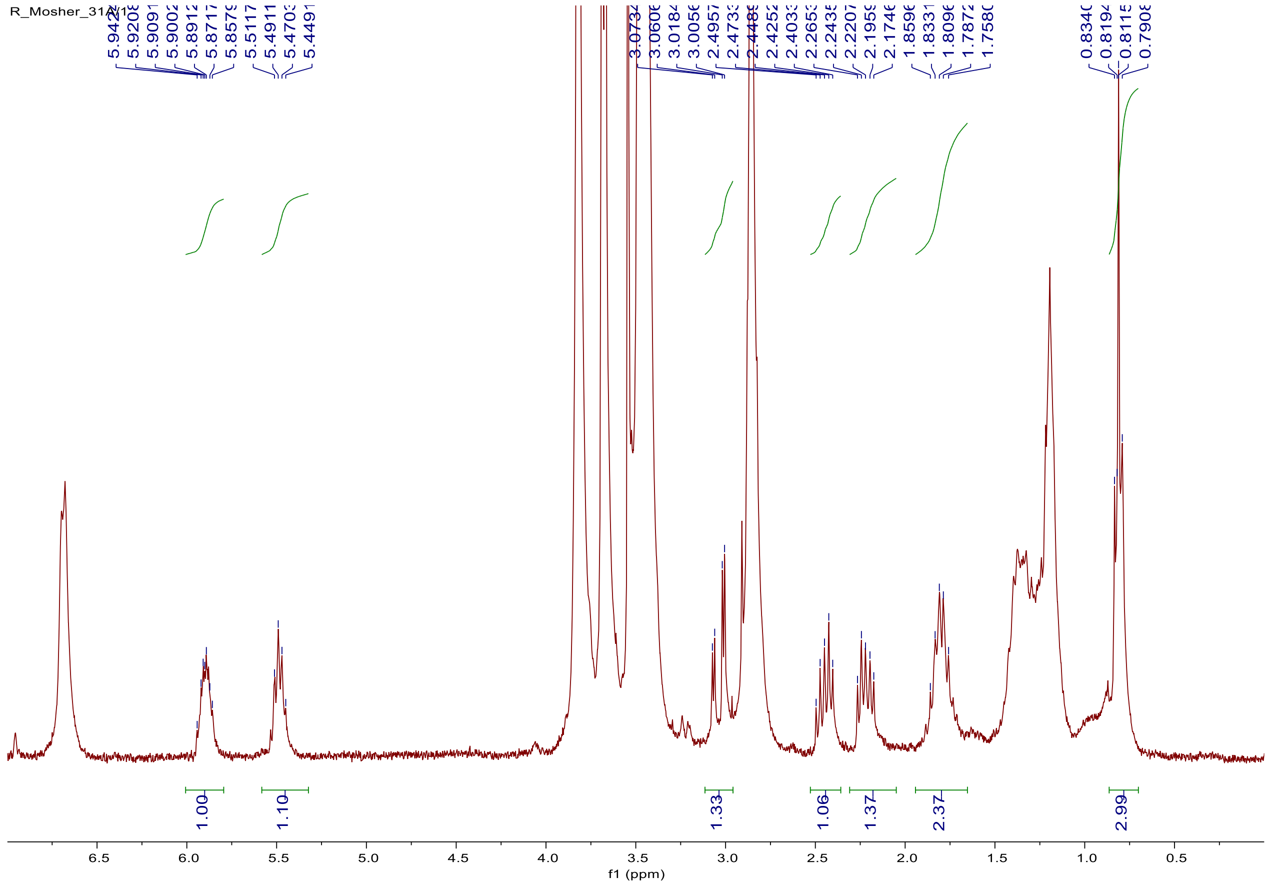

Figure 36. ^{1}H-NMR spectrum of (*S*)-MTPA ester of **3d** (300 MHz, Pyridine-d_5)

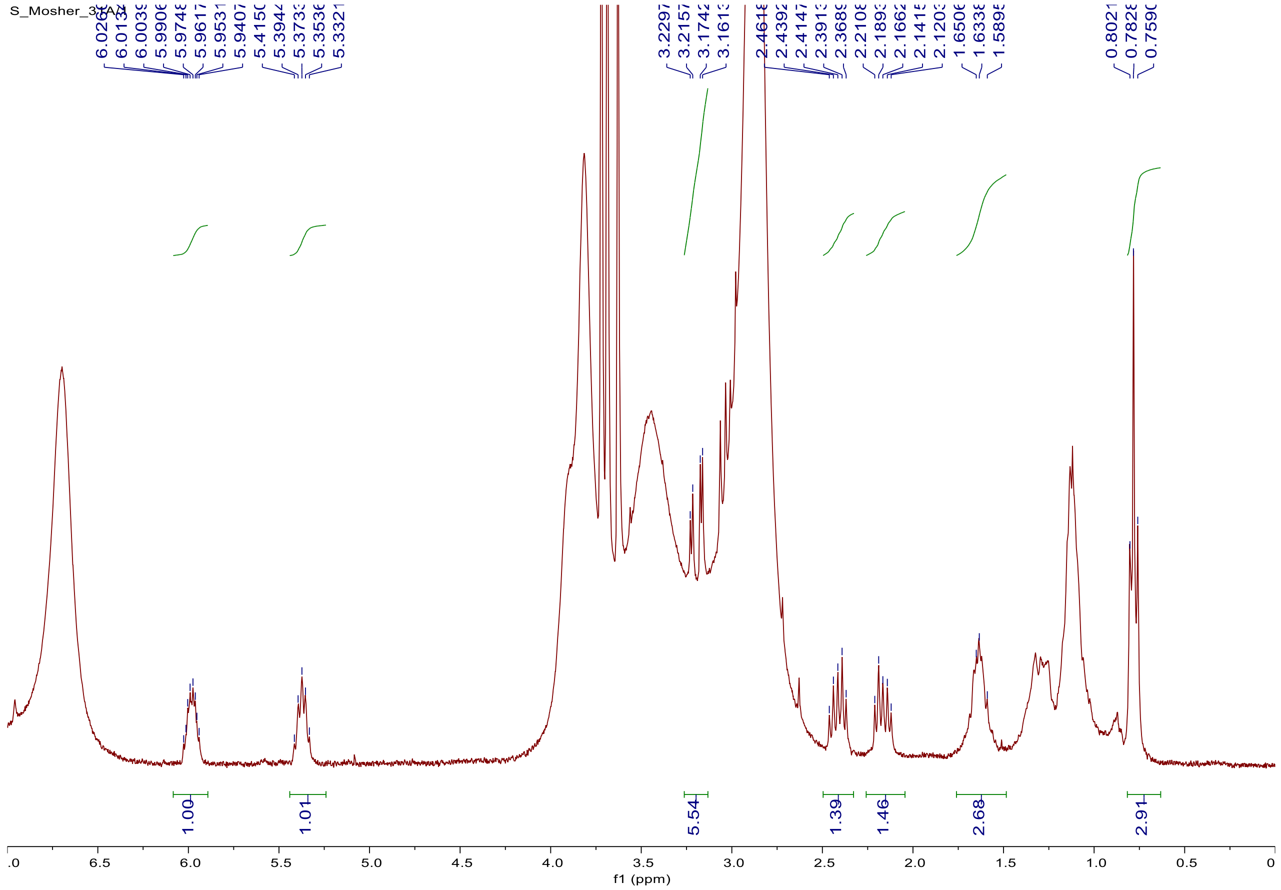

Figure 37. ^{1}H-NMR spectrum of (*R*)-MTPA ester of **3d** (300 MHz, Pyridine-d_5)
